# Correlation between Redox Potential and Solvation Structure in Biphasic Electrolytes for Li Metal Batteries

**DOI:** 10.1002/advs.202203443

**Published:** 2022-10-17

**Authors:** Kyobin Park, Dong‐Min Kim, Kwang‐Ho Ha, Bomee Kwon, Jeonghyeop Lee, Seunghyeon Jo, Xiulei Ji, Kyu Tae Lee

**Affiliations:** ^1^ School of Chemical and Biological Engineering Institute of Chemical Processes Seoul National University 1 Gwanak‐ro, Gwanak‐gu Seoul 08826 Republic of Korea; ^2^ The Molecular Foundry and the Joint Center for Energy Storage Research Department Lawrence Berkeley National Laboratory Berkeley CA 94720 USA; ^3^ Department of Chemistry Oregon State University Corvallis OR 97331‐4003 USA

**Keywords:** batteries, complexation effect, Li^+^ activity, redox potential, solvation structure

## Abstract

The activity of lithium ions in electrolytes depends on their solvation structures. However, the understanding of changes in Li^+^ activity is still elusive in terms of interactions between lithium ions and solvent molecules. Herein, the chelating effect of lithium ion by forming [Li(15C5)]^+^ gives rise to a decrease in Li^+^ activity, leading to the negative potential shift of Li metal anode. Moreover, weakly solvating lithium ions in ionic liquids, such as [Li(TFSI)_2_]^−^ (TFSI = bis(trifluoromethanesulfonyl)imide), increase in Li^+^ activity, resulting in the positive potential shift of LiFePO_4_ cathode. This allows the development of innovative high energy density Li metal batteries, such as 3.8 V class Li | LiFePO_4_ cells, along with introducing stable biphasic electrolytes. In addition, correlation between Li^+^ activity, cell potential shift, and Li^+^ solvation structure is investigated by comparing solvated Li^+^ ions with carbonate solvents, chelated Li^+^ ions with cyclic and linear ethers, and weakly solvating Li^+^ ions in ionic liquids. These findings elucidate a broader understanding of the complex origin of Li^+^ activity and provide an opportunity to achieve high energy density lithium metal batteries.

## Graphical abstract

The chelating of Li^+^ ions with crown ethers, such as [Li(15C5)]^+^ (15C5 = 15‐crown‐5, [Li(15C5)]^+^ = Li^+^‐15C5 complex), lowers the activity of Li^+^ ions in electrolytes, leading to the negative potential shift of Li metal anode. The chelating effect allows the development of innovative high energy density Li metal batteries, such as 3.8 V‐class Li | LiFePO_4_ cells, along with introducing stable biphasic electrolytes.

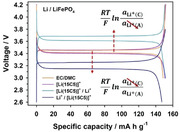

## 1 Introduction

The future of electromobility rests significantly on the advancement of the battery technology toward improving the energy and power densities of rechargeable batteries.^[^ ^1^, ^2^, ^3^, ^4^, ^5^ ^]^ For this reason, great efforts have been devoted to i) developing high specific capacity electrode materials for Li‐ion batteries, including Ni‐rich layered oxide cathode materials^[^ ^6^, ^7^, ^8^, ^9^ ^]^ and Li metal/Si alloy anode materials,^[^ ^10^, ^11^, ^12^, ^13^ ^]^ and ii) exploring new electrochemical systems, such as nonaqueous lithium‐sulfur (Li‐S)^[^ ^14^, ^15^ ^]^ and lithium‐oxygen batteries^[^ ^16^, ^17^ ^]^ and aqueous rechargeable batteries.^[^ ^18^, ^19^, ^20^, ^21^, ^22^ ^]^ However, unfortunately, few electrolytes are suitable for these advanced electrode materials and battery systems because of the narrow electrochemical stability window and poor chemical stability of conventional electrolytes. In this regard, new electrolytes have been intensively investigated to improve the chemical and electrochemical properties of electrolytes.^[^ ^23^, ^24^, ^25^ ^]^ State‐of‐the‐art electrolytes not only serve as a medium that transports Li^+^ ions between electrodes, but also provide functionality that regulates solid electrolyte interphase (SEI) and solvation structures.^[^ ^1^, ^23^, ^26^ ^]^ For example, the electrochemical stability window of water‐in‐salt,^[^ ^18^, ^19^ ^]^ hydrate‐melt,^[^ ^20^ ^]^ and molecular crowding electrolytes^[^ ^21^ ^]^ were improved by regulating the solvation structure of aqueous electrolytes. Localized high concentration electrolytes (LHCE) were introduced to enhance the chemical stability of nonaqueous electrolytes against Li metal under the condition of low Li^+^ ion concentration.^[^ ^27^ ^]^ The cell potential was also controlled by modulating the solvation structures of Li^+^ ions.^[^ ^28^, ^29^ ^]^ In addition, biphasic electrolytes were developed i) to enhance the electrochemical stability window of electrolytes for Li‐ion batteries and ii) to suppress polysulfide crossover in Li‐S batteries.^[^ ^17^, ^22^, ^30^, ^31^, ^32^, ^33^, ^34^ ^]^

Li^+^ activity is a value that varies with surrounding mediums, depending on concentration, ion‐ion interaction, and ion‐solvent interaction.^[^ ^35^ ^]^ The Li^+^ activity in nonaqueous electrolytes, however, was not sufficiently studied because changes in the Li^+^ activity of conventional Li‐ion batteries containing a single‐phase electrolyte do not contribute to regulating the thermodynamics of electrochemical reactions.^[^ ^36^ ^]^ However, when two electrolytes are separated by a Li^+^ ion‐selective membrane, such as biphasic electrolytes, the Nernst equation can be expressed as^[^ ^37^ ^]^

(1) $$E_{\mathrm{Overall}}=E_{\mathrm{Cathode}}-E_{\mathrm{Anode}}=\left(E_{C}^{0}-E_{A}^{0}\right)+\frac{RT}{nF}\ln\frac{a_{{\mathrm{Li}}^{+}\left(C\right)}}{a_{{\mathrm{Li}}^{+}\left(A\right)}}$$

where *E*, *E* ^0^, *n*, *F*, *T*, *R*, and *a* represent cell potential, standard electrode potential, the number of electrons transferred in the reaction, Faraday constant, temperature, gas constant, and activity, respectively (Discussion S1, Supporting Information). In contrast to the single‐phase electrolyte, we can achieve a difference in the activity values of each Li^+^ ion dissolved in phase C and phase A for the biphasic electrolytes. For this reason, overall cell potential can be modulated by the logarithm term of the relative Li^+^ activity ratio between two electrolyte phases.

Herein, we show a significant change in the activity of Li^+^ ions in electrolytes containing the Li^+^ ion complex with cyclic crown ether, 15‐crown‐5, leading to a remarkable decrease in the redox potential of Li metal, compared to conventional carbonate‐based electrolytes. The new redox couple of Li/[Li(15C5)]^+^ (15C5 = 15‐crown‐5, [Li(15C5)]^+^ = Li^+^‐15C5 complex) showed the lower formal potential of −3.32 V (vs NHE) compared to the standard electrode potential of Li/Li^+^, −3.041 V (vs NHE) in aqueous electrolytes. This is due to the complexation effect of Li^+^ chelated with 15‐crown‐5. Moreover, we constructed 3.7 V class Li | LiFePO_4_ cells using the stable biphasic electrolyte using an ion‐selective membrane, in which the electrolytes of anode and cathode sides contained LiPF_6_ in carbonate/dimethyl carbonate (EC/DMC) with and without 15‐crown‐5, respectively. Consequently, the biphasic electrolyte with chelating agents improved the energy density of Li | LiFePO_4_ batteries compared to conventional 3.4 V class Li | LiFePO_4_ containing a single‐phase electrolyte. We also demonstrated a significant increase in the Li^+^ activity in ionic liquid electrolytes containing abundant bis(trifluoromethanesulfonyl)imide (TFSI^−^) anions, leading to a positive potential shift of the LiFePO_4_ cathode due to the formation of a weakly solvating lithium ion structure, such as [Li(TFSI)_2_]^−^. The combination of the complexation effect and the weakly solvation effect further allowed the construction of 3.8 V class Li | LiFePO_4_ cells. Moreover, the relationship between Li^+^ activity, potential shift, and Li^+^ solvation structure was investigated by examining chelated Li^+^ ions with cyclic/linear ethers and weakly solvated Li^+^ ions with ionic liquids. More stabilization of Li^+^ complex gave rise to a decrease in the activity of Li^+^ in electrolytes, whereas weakly solvating Li^+^ ions increased the activity of Li^+^ in electrolytes, eventually resulting in a significant increase in cell potential. These findings provide an opportunity to design advanced electrolytes for high energy density Li metal batteries.

## 2 Negative Potential Shift of the Li Metal Anode

We constructed three electrochemical cells, each consisting of a LiFePO_4_ cathode and a Li metal anode, as below:

(I) Li | Li^+^, PF_6_^−^ | LiFePO_4_
(II) Li | [Li(15C5)]^+^, PF_6_^−^ | LiFePO_4_
(III) Li | [Li(15C5)]^+^, PF_6_^−^ || Li^+^, PF_6_^−^ | LiFePO_4_

The first cell (I) has a single‐phase electrolyte, such as 1 m LiPF_6_ in EC/DMC (1/1, v/v), which is denoted as the conventional cell. The second cell (II), denoted as the [Li(15C5)]^+^ cell, also has a single‐phase electrolyte, such as 0.5 m LiPF_6_ in EC/DMC/15C5 (2/2/1, v/v/v), in which the molar ratio of 15C5 to Li^+^ is 2:1. The third cell (III), denoted as the [Li(15C5)]^+^/Li^+^ cell, contains the biphasic electrolytes separated by a Li^+^ ion‐selective Nafion membrane. The electrolyte of the LiFePO_4_ cathode side was 0.5 m LiPF_6_ in EC/DMC, whereas that of the Li metal anode side was 0.5 m LiPF_6_ in EC/DMC/15C5. Crown ethers are known to form complexes with lithium cations, such as [Li(15C5)]^+^, and to homogenize Li^+^ flux, leading to uniform electroplating of Li metal.^[^ ^38^, ^39^ ^]^ Home‐made cells (Figure S1, Supporting Information) and 2032‐type coin cells were used for biphasic electrolytes and single‐phase electrolytes, respectively. We also prepared the Li^+^ ion‐selective Nafion membrane through ion‐exchange between H^+^ and Li^+^ (Figure S2, Supporting Information).

To verify the impermeability of the Li^+^ ion‐selective Nafion membrane to 15C5 in the biphasic electrolyte, we built 2‐chamber side diffusion cells with the Li^+^ ion‐selective Nafion membrane where the donor chamber was filled with 0.5 m LiPF_6_ in EC/DMC and the receptor one was filled with 0.5 m LiPF_6_ in EC/DMC/15C5. Solutions were retrieved from the donor chamber after various periods of time, and then the membrane permeability to 15C5 was estimated using ^1^H and ^13^C nuclear magnetic resonance (NMR) spectroscopy (Figure S3, Supporting Information). The characteristic peaks of 15C5 were not observed in the solutions of the donor chamber even after one week. We also examined changes in the Li^+^ ion concentration of the solutions in the donor and receptor chambers using inductively coupled plasma atomic emission spectrometer (ICP‐AES) (Table S1, Supporting Information). The concentrations of Li^+^ ions in both chambers remained almost unchanged for one week. These results imply that the crossover of 15C5 through the membrane was insignificant.

**Figure 1:**
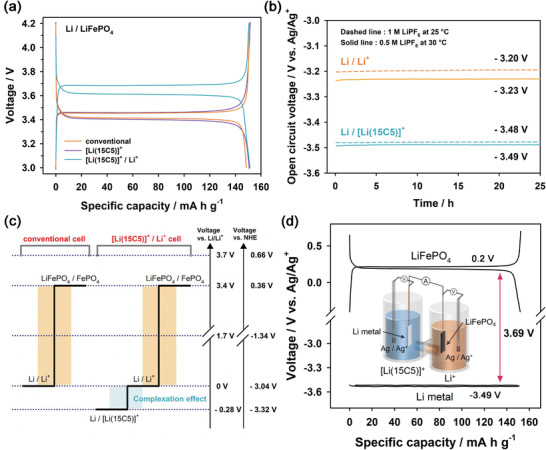
a) Voltage profiles of i) conventional cell, ii) [Li(15C5)]^+^ cell, and iii) [Li(15C5)]^+^/Li^+^ cell at a 0.5C rate and 30 °C. b) OCV profiles of Li metal electrodes each with i) 0.5 m LiPF_6_ in EC/DMC and 0.5 m LiPF_6_ in EC/DMC/15C5 at 30 °C (solid line) and each with ii) 1 m LiPF_6_ in EC/DMC and 1 m LiPF_6_ in EC/DMC/15C5 at 25 °C (dashed line). Cells with LiPF_6_ in EC/DMC and LiPF_6_ in EC/DMC/15C5 are denoted as Li/Li^+^ and Li/[Li(15C5)]^+^, respectively. OCV was measured using the Ag/Ag^+^ reference electrode. c) Schematic illustration for the increased cell potential of the [Li(15C5)]^+^/Li^+^ cell compared to the conventional cell. d) Voltage profiles of LiFePO_4_ and Li metal electrodes obtained using the four‐electrode cell at a 0.5C rate and 30 °C for the measurement of a liquid junction potential (inset: illustration of a four‐electrode cell configuration).

**Figure** **1**a compares the voltage profiles of the conventional, [Li(15C5)]^+^, and [Li(15C5)]^+^/Li^+^ cells at a 0.5C rate and 30 °C. The conventional and [Li(15C5)]^+^ cells, both having a single‐phase electrolyte, showed the same voltage profiles with a cell potential of ≈3.4 V, regardless of the composition of electrolytes. This is due to the fact that the solvation energies and desolvation energies of Li^+^ ions are cancelled when a single‐phase electrolyte is used. The cell potential of the [Li(15C5)]^+^/Li^+^ cell was, however, ≈3.7 V, which was significantly higher than those of the conventional and [Li(15C5)]^+^ cells. The open‐circuit voltage (OCV) of the [Li(15C5)]^+^/Li^+^ cell was 3.67 V at the state of charge (SOC) of 50% (Figure S4, Supporting Information). To clarify the origin of the potential shift in the [Li(15C5)]^+^/Li^+^ cell, we compared the redox potentials of Li/Li^+^ and Li/[Li(15C5)]^+^ couples using the Ag/Ag^+^ reference electrode (Figure 1b). The Ag/Ag^+^ reference electrode was calibrated with reference to the ferrocene/ferrocenium ion couple (Figure S5, Supporting Information). Whereas the equilibrium potential of the Li/Li^+^ was ≈−3.23 and −3.20 (vs Ag/Ag^+^) for 0.5 m LiPF_6_ at 30 °C and 1 m LiPF_6_ at 25 °C (standard state), respectively, the Li/[Li(15C5)]^+^ redox couple showed the equilibrium potential of −3.49 and −3.48 V (vs Ag/Ag^+^) each under the same conditions as Li/Li^+^, respectively. The equilibrium potential difference between Li/Li^+^ and Li/[Li(15C5)]^+^ couples was almost the same as the potential shift in the [Li(15C5)]^+^/Li^+^ cell compared to the conventional cell (Figure S4 and S6, Supporting Information). This is also supported by the voltage profile of a symmetric cell for Li | [Li(15C5)]^+^, PF_6_^−^ || Li^+^, PF_6_^−^ | Li under the condition of an areal capacity of 0.5 mA h cm^−2^ at a current density of 0.5 mA cm^−2^ (Figure S7, Supporting Information). The equilibrium potential for the plating and stripping of Li metal was ≈−0.25 V, which was coincident with the potential difference between the Li/Li^+^ and Li/[Li(15C5)]^+^ redox couples. Therefore, these results supported that the increased cell potential of the [Li(15C5)]^+^/Li^+^ cell were due to a negative potential shift of Li metal anode, as shown in Figure 1c. Moreover, this reveals that the formal potential of the Li/[Li(15C5)]^+^ couple is −3.32 V (vs NHE). The correlation between the redox potentials of Li/Li^+^, Ag/Ag^+^, and NHE references was detailed in Discussion S2 in the Supporting Information. In the same manner, when we switched electrolytes for each other in the biphasic electrolyte of the [Li(15C5)]^+^/Li^+^ cell as below, the cell potential was rather decreased to ≈3.2 V (Figure S8, Supporting Information). This was due to a negative potential shift of the LiFePO_4_ cathode:

(IV) Li | Li^+^, PF_6_^−^ || [Li(15C5)]^+^, PF_6_^−^ | LiFePO_4_

In addition, we measured a liquid junction potential of the biphasic electrolyte in the [Li(15C5)]^+^/Li^+^ cell using the four‐electrode cell configuration consisting of a Li metal anode, a LiFePO_4_ cathode, and two Ag/Ag^+^ reference electrodes (Figure 1d). The redox potential values of the LiFePO_4_/FePO_4_ and Li/Li^+^ redox couples in the four‐electrode cell were ≈+0.20 and −3.49 V (vs Ag/Ag^+^) at 30 °C, respectively. A difference in their redox potentials (3.69 V) was almost the same as the cell potential of the [Li(15C5)]^+^/Li^+^ cell (3.67 V), implying that the liquid junction potential of the biphasic electrolyte was ≈0.02 V, which is negligibly small (Discussion S3, Supporting Information).

**Figure 2:**
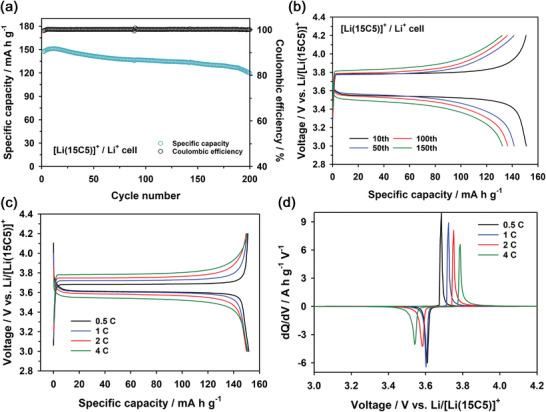
a) Cycle performance and b) the corresponding voltage profiles of [Li(15C5)]^+^/Li^+^ cell consisting of LiFePO_4_ cathode and Li metal anode at a 4C rate. c) Voltage profiles and d) the corresponding d*Q*/d*V* plots of the [Li(15C5)]^+^/Li^+^ cell consisting of LiFePO_4_ cathode and Li metal anode at various C‐rates.

**Figure** **2**a,b shows the cycle performance and corresponding voltage profiles of the [Li(15C5)]^+^/Li^+^ cell for LiFePO_4_ at a 4C rate and 30 °C, respectively. The [Li(15C5)]^+^/Li^+^ cell showed excellent cycle performance, such as stable capacity retention over 200 cycles, without a change in the equilibrium cell potential during cycling. This reveals that the biphasic electrolyte was stable, suppressing the crossover of 15C5 during cycling. This is also supported by changes in the Li^+^ ion concentration on each phase of the biphasic electrolyte during charge and discharge (Figure S9, Supporting Information). The Li^+^ ion concentrations on both phases of the biphasic electrolyte remained almost unchanged during charge and discharge. We also examined the cycle performances of [Li(15C5)]^+^/Li^+^ cells for LiFePO_4_ using various amounts of electrolytes per capacity at a 4C rate and 30 °C (Figure S10, Supporting Information). The [Li(15C5)]^+^/Li^+^ cell showed more stable capacity retention as the amount of electrolytes increased. This is attributable to the fact that the electrolytes of LiPF_6_ in carbonate solvents are vulnerable to Li metal. Figure S11 in the Supporting Information shows the Nyquist plots of the [Li(15C5)]^+^/Li^+^ cell at fully charged (4.2 V) and discharged (3 V) states for various cycle numbers. The size of semi‐circles increased with increasing cycle number. This implies that the interfacial overpotential due to charge‐transfer resistance and SEI resistance increased with increasing cycle number, leading to a gradual increase in the cell overpotential during cycling (Figure 2b). Moreover, we observed that an additional large semi‐circle appeared in the Nyquist plots after 100 cycles. This is attributable to SEI resistance due to electrolyte decomposition that has accumulated on the Li metal surface during cycling. The voltage profiles and d*Q*/d*V* profiles of the [Li(15C5)]^+^/Li^+^ cell at various C‐rates were also presented in Figure 2c,d.

The same behavior of the [Li(15C5)]^+^/Li^+^ cell was observed when we replaced LiFePO_4_ with LiCoO_2_ in the [Li(15C5)]^+^/Li^+^ cell as follows:

(V) Li | Li^+^, PF_6_^−^ | LiCoO_2_
(VI) Li | [Li(15C5)]^+^, PF_6_^−^ || Li^+^, PF_6_^−^ | LiCoO_2_

**Figure 3:**
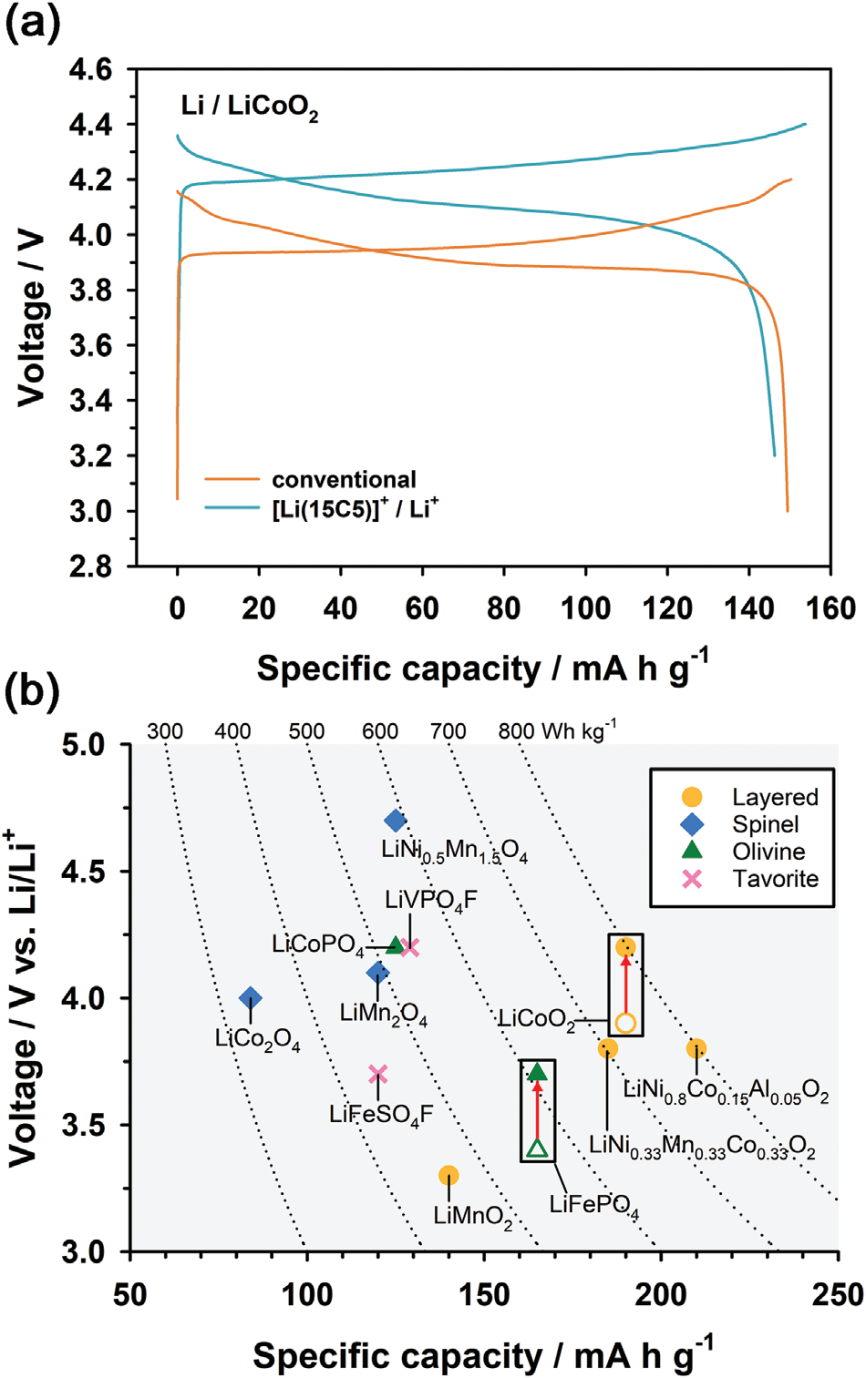
a) Voltage profiles of i) Li | 1 m LiPF_6_ in EC/DMC | LiCoO_2_ (conventional cell) and ii) Li | 0.5 m LiPF_6_ in EC/DMC/15C5 ‖ 0.5 m LiPF_6_ in EC/DMC | LiCoO_2_ ([Li(15C5)]^+^/Li^+^ cell) cells at a 0.5C rate and 30 °C. b) Theoretical specific energy (average operating voltage × specific capacity) of various cathode materials.

**Figure** **3**a compares the voltage profiles of the conventional cell (V) and [Li(15C5)]^+^/Li^+^ (VI) cells for LiCoO_2_ at a 0.5C rate and 30 °C. The voltage profile of the [Li(15C5)]^+^/Li^+^ cell for LiCoO_2_ shifted upward by ≈0.3 V compared to that of the conventional cell for LiCoO_2_, which was consistent with the results for LiFePO_4_. Assuming that the cell potentials of LiFePO_4_ and LiCoO_2_ increase by 0.3 V without changes in their specific capacity, the energy densities of LiFePO_4_ and LiCoO_2_ cathodes theoretically increase from 561 and 741 to 610 and 798 Wh kg^−1^, respectively (Figure 3b). This suggests that we can achieve an energy density increase of ≈8–9% albeit without changes in electrode materials. Detailed cell parameters for the calculation of energy density were presented in Table S2 (Supporting Information).

**Figure 4:**
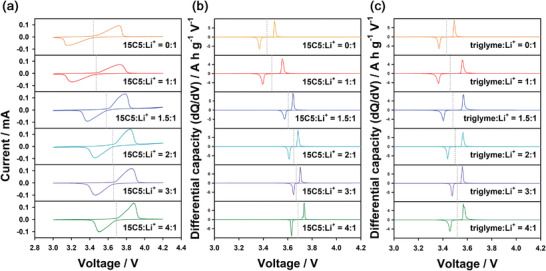
a) Cyclic voltammograms (scan rate = 0.1 mV s^−1^) and b) d*Q*/d*V* plots (0.5C rate) of Li | 0.5 m LiPF_6_ in EC/DMC/15C5 ‖ 0.5 m LiPF_6_ in EC/DMC | LiFePO_4_ cells for various molar ratios of 15C5 to Li^+^. c) d*Q*/d*V* plots (0.5C rate) of Li | 0.5 m LiPF_6_ in EC/DMC/triglyme ‖ 0.5 m LiPF_6_ in EC/DMC | LiFePO_4_ cells for various molar ratios of triglyme to Li^+^.

**Figure 5:**
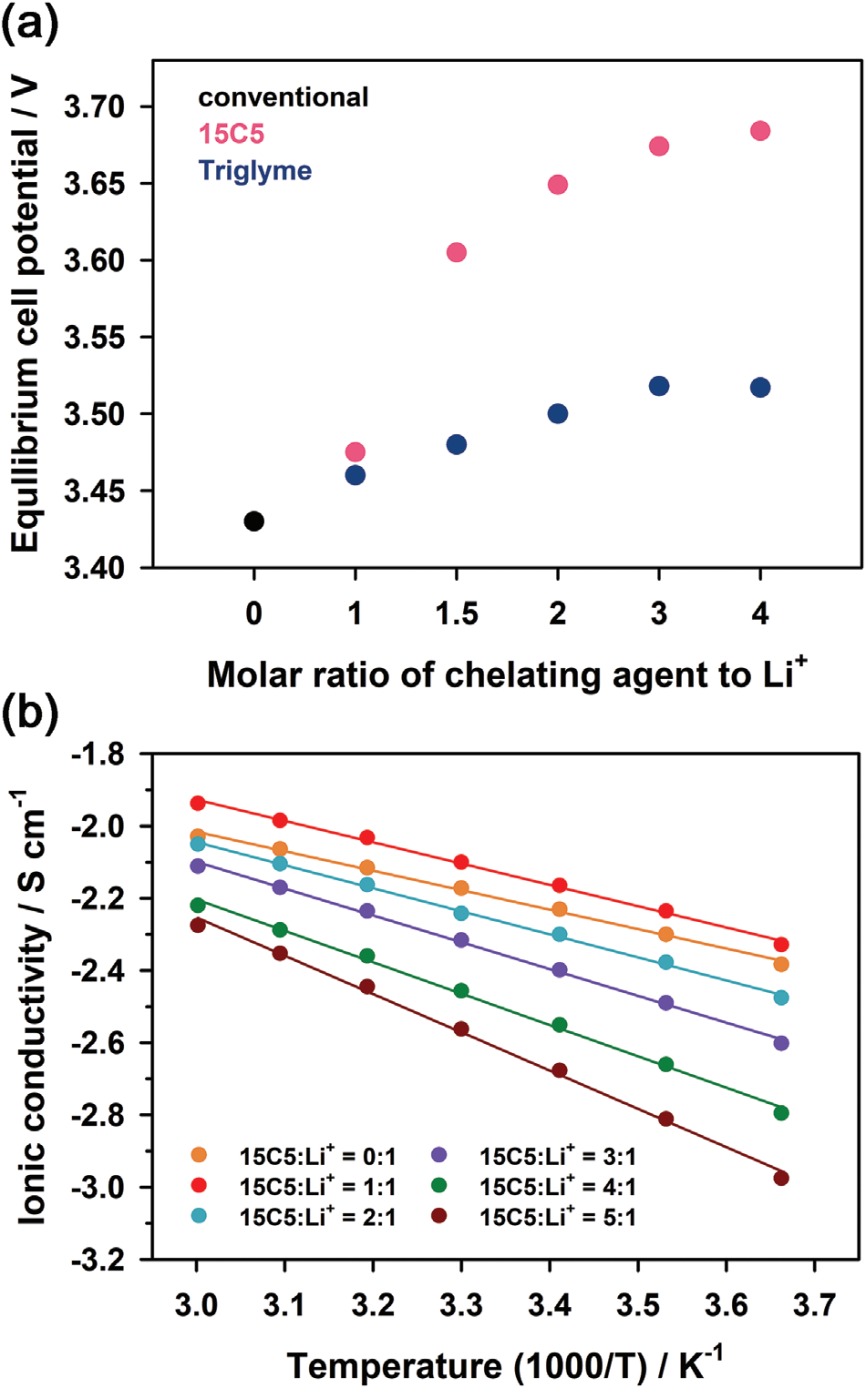
a) Equilibrium potential values of [Li(15C5)]^+^/Li^+^ and [Li(triglyme)]^+^/Li^+^ cells consisting of LiFePO_4_ cathode and Li metal anode for various molar ratios of chelating agent to Li^+^. Equilibrium potentials were determined as the average values of cathodic and anodic peak potentials in the d*Q*/d*V* profiles. b) Ionic conductivity of 0.5 m LiPF_6_ in EC/DMC/15C5 as a function of temperature for various molar ratios of 15C5 to Li^+^.

We compared changes in the cell potential of [Li(15C5)]^+^/Li^+^ cells containing Li metal and LiFePO_4_ at various molar ratios of chelating agent to Li^+^ to demonstrate the role of chelating agents in the potential shift of Li metal. **Figure** **4**a,b shows the cyclic voltammogram and d*Q*/d*V* profiles of [Li(15C5)]^+^/Li^+^ cells for Li | LiFePO_4_ at various molar ratios of 15C5 to Li^+^. The cell potential of [Li(15C5)]^+^/Li^+^ cells increased with the molar ratio of 15C5 to Li^+^, implying that the activity of Li^+^ ($a_{{\mathrm{Li}}^{+}\left(A\right)}$) in the electrolyte of Li metal side decreased with increasing the number of 15C5 chelating with Li^+^ (**Figure** **5**a). The chelation effect is known to show the greater stability of chelated complexes compared to their nonchelated (solvated) analogues largely because of increases in free solvent molecules.^[^ ^40^ ^]^ This implies that the thermodynamic stability of the solvation structure increased as the entropy of the electrolyte increased.^[^ ^26^, ^41^ ^]^ Therefore, a decrease in the activity of Li^+^ in the electrolytes containing 15C5 was due to the improved stabilization of [Li(15C5)]^+^ complexes.

The negative potential shift of Li metal can be demonstrated in terms of an equilibrium between Li^+^ and 15C5. The equilibrium at the anode side is expressed as^[^ ^37^ ^]^

(2) $${\mathrm{Li}}^{+}+15C5⇌{\left[\mathrm{Li}\left(15C5\right)\right]}^{+}\left(K_{f}=\frac{a_{{\left[\mathrm{Li}\left(15C5\right)\right]}^{+}}}{a_{{\mathrm{Li}}^{+}\left(A\right)}a_{15C5}}\right)$$

where *K*_f_ is a formation constant of chelated Li^+^ ions from solvated Li^+^ ions. Since *K*_f_ is a constant, each concentration of [Li(15C5)]^+^ and Li^+^ increases and decreases, respectively, with increasing that of 15C5. This implies that the activity of solvated Li^+^ is higher than that of chelated Li^+^ with 15C5, because the activity of Li^+^ ($a_{{\mathrm{Li}}^{+}\left(A\right)}$) in the electrolyte of Li metal side decreased with increasing the concentration of [Li(15C5)]^+^. Since the redox potential of the Li/[Li(15C5)]^+^ was 0.28 V higher than that of the Li/Li^+^, Equation (1) shows $\frac{RT}{nF}\ln\frac{a_{{\mathrm{Li}}^{+}\left(C\right)}}{a_{{\mathrm{Li}}^{+}\left(A\right)}}=0.28\;V$ under a standard condition. This reveals that the Li^+^ activity in the conventional carbonate‐based electrolyte without 15C5 ($a_{{\mathrm{Li}}^{+}\left(C\right)}$) was ≈5.5 × 10^4^ times higher than that in the electrolyte containing 15C5 ($a_{{\mathrm{Li}}^{+}\left(A\right)}$) (Discussion S4, Supporting Information). The Li^+^ activity values indicate the average activity of all species of Li^+^ cations (solvated and chelated Li^+^ cations) in electrolytes.

Moreover, we examined linear triethylene glycol dimethyl ether (triglyme) as a complexing agent, leading to the formation of [Li(triglyme)]^+^ complex (Figure 4c and Figure 5a). [Li(triglyme)]^+^ showed the same behavior as [Li(15C5)]^+^. The cell potential of [Li(triglyme)]^+^/Li^+^ cells for Li | LiFePO_4_ increased, as the molar ratio of triglyme to Li^+^ increased. However, the cell potentials of [Li(triglyme)]^+^/Li^+^ cells were lower than those of [Li(15C5)]^+^/Li^+^ cells at the same molar ratio of chelating agent to Li^+^. This reveals that the Li^+^ activity in the electrolyte containing 15C5 was lower than that in the electrolyte containing triglyme at the same concentration of chelating agents. This behavior is demonstrated in terms of the macrocyclic effect that cyclic ligands stabilize the complexes more than linear ligands^[^ ^42^, ^43^ ^]^ (Discussion S5, Supporting Information). This implies that the potential shift due to a change in Li^+^ activity depend on the stabilization degree of the complexes.

In addition, Figure 5b shows the ionic conductivity of 0.5 m LiPF_6_ in EC/DMC/15C5 (1/1/*x*, 0 ≤ *x* ≤ 2, v/v/v) for various molar ratios of 15C5 to Li^+^ as a function of temperature. The ionic conductivity of the electrolytes containing 15C5 decreased with increasing the ratio of 15C5 to Li^+^. This is probably because the viscosity of the electrolytes increased with the ratio of 15C5 to Li^+^ (Table S3, Supporting Information). We also compared the electrochemical stability window of 0.5 m LiPF_6_ in EC/DMC (1/1, v/v) and 0.5 m LiPF_6_ in EC/DMC/15C5 (2/2/1, v/v/v), as shown in their linear sweep voltammograms (LSV) (Figure S12, Supporting Information). This reveals that no drastic cathodic decomposition was observed in both electrolytes prior to each Li metal plating.

**Figure 6:**
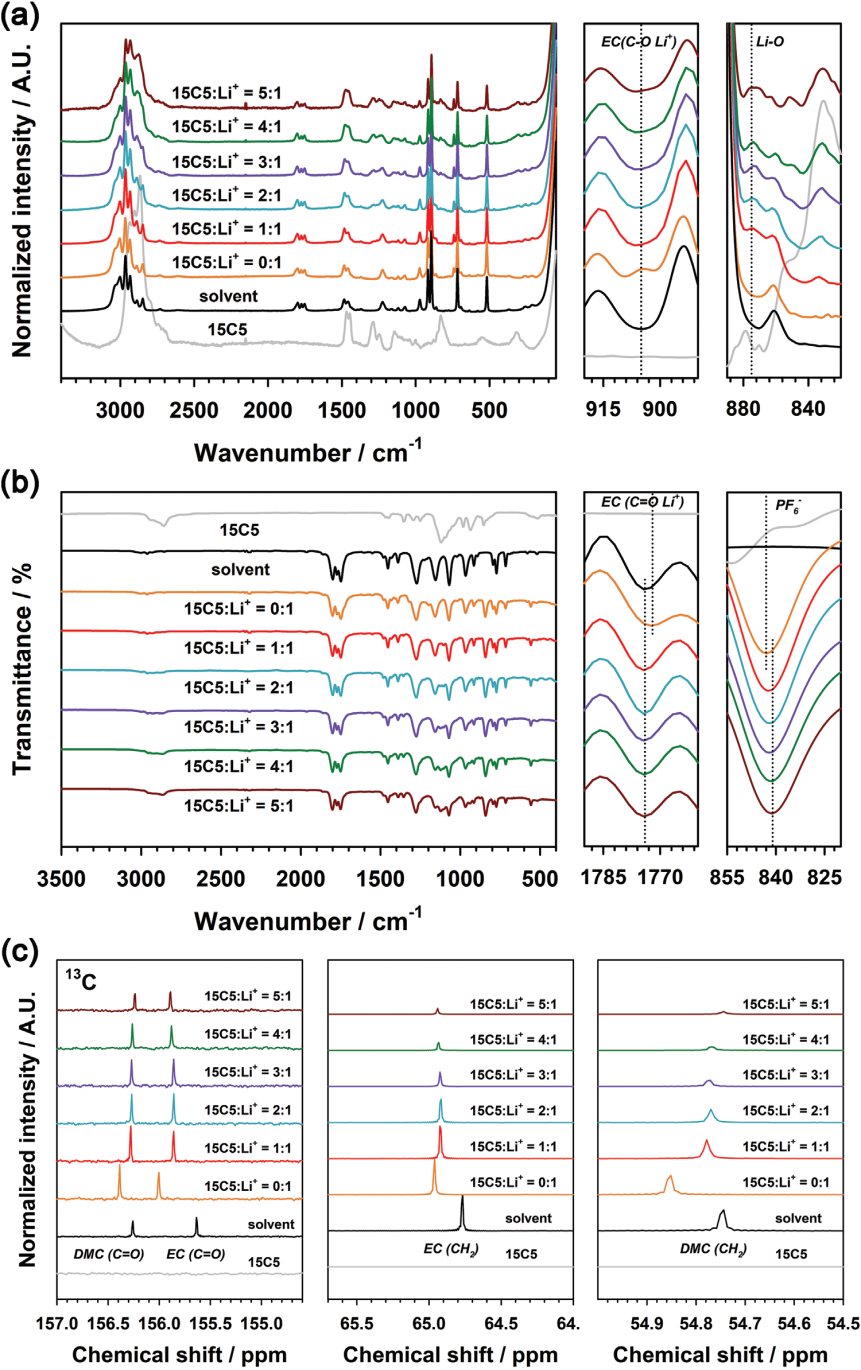
a) Raman, b) FT‐IR, and c) ^13^C NMR spectra of pure solvents and the electrolytes of 0.5 m LiPF_6_ in EC/DMC/15C5 for various molar ratios of 15C5 to Li^+^.

To elucidate the role of solvation structures in Li^+^ activity changes, the solvation structures of Li^+^ in 0.5 m LiPF_6_ in EC/DMC/15C5 for various molar ratios of 15C5 to Li^+^ were examined using Raman, FT‐IR, and ^13^C NMR spectroscopies. The characteristic vibration frequencies and chemical shifts of electrolytes were summarized in Tables S4–S6 in the Supporting Information. **Figure** **6**a shows changes in the Raman spectra of the electrolytes with increasing the molar ratio of 15C5 to Li^+^. In the pristine electrolyte containing no 15C5, EC is known to solvate Li^+^, giving rise to a distinguishable peak at 905 cm^−1^ for Li^+^∙∙∙O—C (EC).^[^ ^44^ ^]^ The peak at 905 cm^−1^ disappeared and a new peak appeared at 875 cm^−1^ after adding 15C5 to the pristine electrolyte. Moreover, the peak intensity at 875 cm^−1^ gradually increased with increasing the molar ratio of 15C5 to Li^+^. This implies that Li^+^ prefers to complex with 15C5 rather than to be solvated by EC because the peak at 875 cm^−1^ corresponds to Li^+^∙∙∙O—C (ether). The complexation of Li^+^ with 15C5 was also supported by the FT‐IR spectra of the electrolytes (Figure 6b). The peak of Li^+^∙∙∙O=C (EC) at 1772 cm^−1^ shifted to 1774 cm^−1^, which is the wavenumber of pure EC, after adding 15C5 to the pristine electrolyte. This reveals that the ion‐solvent interaction between Li^+^ and EC was weakened because of the formation of [Li(15C5)]^+^ complexes. This behavior was also coincident with the fact that the P—F stretch peak of PF_6_^−^ shifted from 843 to 841 cm^−1^ after the addition of 15C5. The formation of [Li(15C5)]^+^ complexes gave rise to weakening the cation–anion interaction, leading to a peak shift to a lower wavenumber.^[^ ^45^, ^46^ ^] 13^C NMR spectra also show the solvation structures of [Li(15C5)]^+^ with EC and DMC (Figure 6c). When we compared the NMR spectra of the mixed solvent of EC/DMC with and without LiPF_6_, the ^13^C chemical shift of EC was more deshielded than that of DMC. This implies that Li^+^ was preferentially coordinated with EC, such as Li^+^(EC)_4_, rather than with DMC. For this reason, the ^13^C chemical shift of EC was rather shielded by the addition of 15C5 to the electrolytes containing LiPF_6_, as Li^+^ ions were desolvated from Li^+^(EC)_4_ and chelated with 15C5, forming [Li(15C5)]^+^.

## 3 Positive Potential Shift of the LiFePO_4_ Cathode

In the same manner, we introduced weakly solvating electrolytes at the cathode side to increase the activity of Li^+^ ions in electrolytes ($a_{{\mathrm{Li}}^{+}\left(C\right)}$), thus increasing the redox potential of the cathode. We constructed two electrochemical cells each consisting of a LiFePO_4_ cathode and a Li metal anode, as below:

(VII) Li | 0.5 m LiTFSI in EC/DMC || 0.5 m LiTFSI in EMIM‐TFSI | LiFePO_4_
(VIII) Li | 0.5 m LiTFSI in EC/DMC || 0.5 m LiTFSI in HMIM‐TFSI | LiFePO_4_

**Figure 7:**
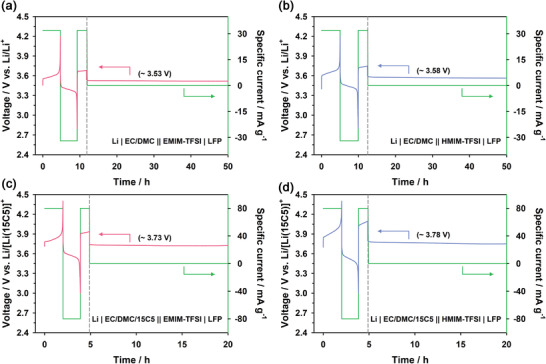
OCV profiles of a) Li | 0.5 m LiTFSI in EC/DMC ‖ 0.5 m LiTFSI in EMIM‐TFSI | LiFePO_4_, b) Li | 0.5 m LiTFSI in EC/DMC ‖ 0.5 m LiTFSI in HMIM‐TFSI | LiFePO_4_, c) Li | 0.5 m LiTFSI in EC/DMC/15C5 ‖ 0.5 m LiTFSI in EMIM‐TFSI | LiFePO_4_ and d) Li | 0.5 m LiTFSI in EC/DMC/15C5 ‖ 0.5 m LiTFSI in HMIM‐TFSI | LiFePO_4_ at the SOC level of 50% and 30 °C. OCV profiles were obtained after one cycle at a, b) 0.2C and c,d) 0.5C rates.

1‐ethyl‐3‐methylimidazolium bis(trifluoromethylsulfonyl)imide (EMIM‐TFSI) and 1‐hexyl‐3‐methylimidazolium bis(trifluoromethanesulfonyl)imide (HMIM‐TFSI) were used as electrolytes to form Li^+^‐TFSI^−^ ion pairs in electrolytes because the Li^+^‐TFSI^−^ coordination is known to lower the solvating power of solvents.^[^ ^47^ ^]^ Both electrolytes also showed high oxidation stability, as shown in their LSV profiles (Figure S13, Supporting Information). **Figure** **7**a,b and Figure S14 in the Supporting Information show the OCV profiles for the cells of (VII) and (VIII) with 0.5 and 1.0 m LiTFSI electrolytes, respectively, at the SOC level of 50%. The cell potential of the cell (VII) increased by 0.13 and 0.16 V for 0.5 m LiTFSI and 1 m LiTFSI, respectively, at 30 °C compared to that of the conventional cell. The cell potential of the cell (VIII) also increased by 0.18 and 0.2 V for 0.5 m LiTFSI and 1 m LiTFSI, respectively. Moreover, we constructed 3.8 V class Li | LiFePO_4_ cells through the combination effect of the negative potential shift of Li metal anode and the positive potential shift of LiFePO_4_ cathode, as below:

(IX) Li | 0.5 m LiTFSI in EC/DMC/15C5 (2/2/1) || 0.5 m LiTFSI in EMIM‐TFSI | LiFePO_4_
(X) Li | 0.5 m LiTFSI in EC/DMC/15C5 (2/2/1) || 0.5 m LiTFSI in HMIM‐TFSI | LiFePO_4_

Figure 7c,d shows the OCV profiles for the cells of (IX) and (X) at the SOC level of 50%. The cells of (IX) and (X) showed the equilibrium potentials of 3.73 and 3.78 V, respectively. The potential shift of the cell (X) compared to the conventional cell was almost the same as the OCV of the Li/Li symmetric cell for Li | 0.5 m LiTFSI in EC/DMC/15C5 ‖ 0.5 m LiTFSI in HMIM‐TFSI | Li in Figure S15 in the Supporting Information.

**Figure 8:**
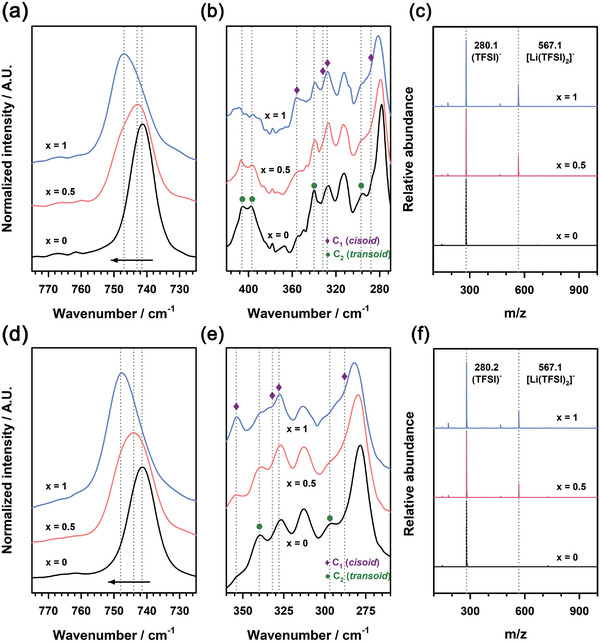
a,b) Raman and c) ESI‐MS spectra of the electrolyte solutions for *x* m LiTFSI in EMIM‐TFSI (*x* = 0, 0.5, and 1). d,e) Raman and f) ESI‐MS spectra of the electrolyte solutions for *x* m LiTFSI in HMIM‐TFSI (*x* = 0, 0.5, and 1).

The solvation structures of Li^+^ ions in ionic liquid‐based electrolytes were examined using Raman spectroscopy and electrospray‐ionization mass spectrometry (ESI‐MS) to demonstrate the role of solvation structures in increases in Li^+^ activity for electrolytes at the cathde side. **Figure** **8**a,d shows changes in the Raman spectra of the electrolytes with increasing the concentration of LiTFSI. The peak of free TFSI^−^ at 741.5 cm^−1^ shifted gradually to higher wavenumbers for both EMIM‐TFSI and HMIM‐TFSI with increasing the concentration of LiTFSI from 0 to 1 m. This reveals that the peak shift is attributed to the coordination of TFSI^−^ anions with Li^+^ ions. This coordination gave rise to the formation of weakly solvating Li^+^ ions, leading to increases in the activity of Li^+^ in ionic liquid‐based electrolytes. The coordination of Li^+^ with TFSI^−^ in the electrolytes was also supported by the Raman spectra of the *cis* and *trans* conformers of TFSI^−^ (Figure 8b,e). In equilibrium, TFSI^−^ anions coexist in *cis* and *trans* forms, in which the *trans* conformer is ≈2.2 kJ mol^−1^ more stable than the *cis* conformer.^[^ ^48^ ^]^ The peak intensities of *trans* TFSI^−^ and *cis* TFSI^−^ decreased and increased, respectively, gradually with increasing the concentration of LiTFSI. This implies that *cis* conformers were preferred over *trans* conformers as TFSI^−^ coordinated with Li^+^. In addition, ESI‐MS spectra showed the solvation sheath structures of TFSI^−^ anions coordinated with Li^+^ (Figure 8c,f). The peaks at the *m*/*z* of 567.1 and 280.1 correspond to [Li(TFSI)_2_]^−^ and free TFSI^−^ anions, implying that Li^+^ ions were coordinated with TFSI^−^ anions.

## 4 Conclusion

We demonstrated that the simple replacement of a single‐phase electrolyte with a biphasic electrolyte gave rise to a significant increase in cell potential by lowering and raising the redox potentials of the Li metal anode and the LiFePO_4_ cathode, respectively. This strategy is not only limited to Li | LiFePO_4_ cells, but also applicable to other cathode materials, such as LiCoO_2_ and Ni‐rich layered oxides. This implies that we can improve the energy density of Li metal‐ion batteries for all cathode materials, regardless of the redox potential of cathode materials.

We also clarified the correlation between activity, cell potential, and solvation structure. The role of the complexation effect in changes in Li^+^ activity was examined using carbonate solvent‐based nonaqueous electrolytes containing cyclic and linear chelating agents, such as 15C5 and triglyme. The activity of Li^+^ chelated with 15C5 in the electrolyte of LiPF_6_ in EC/DMC was approximately five orders of magnitude lower than that of Li^+^ solvated with carbonate solvents in the conventional electrolyte of LiPF_6_ in EC/DMC without additives. This was due to the chelation and macrocyclic effect of Li^+^ complexes. A decrease in Li^+^ activity led to the negative potential shift of Li/[Li(15C5)]^+^ and Li/[Li(triglyme)]^+^ redox couples for Li metal electrodes. Li/[Li(15C5)]^+^ showed a significantly low formal potential of −3.32 V (vs NHE). In addition, the activity of Li^+^ coordinated with TFSI^−^ in the ionic liquid‐based electrolytes increased with increasing the concentration of LiTFSI. This was attributed to the formation of weakly solvating Li^+^ ions, such as [Li(TFSI)_2_]^−^ in ionic liquids. Increases in Li^+^ activity led to the positive potential shift of the LiFePO_4_ cathode. Eventually, the combination effect of the negative potential shift of Li metal anode and the positive potential shift of LiFePO_4_ cathode allows the development of 3.8 V class Li | LiFePO_4_ along with introducing the biphasic electrolytes with a stable ion‐selective membrane. This suggests that an improved understanding of correlation between activity and solvation structure will provide insights on designing new functional electrolytes to improve the energy density of Li metal batteries. However, since the ion‐selective Nafion membrane is not considered to be permanently stable, long‐term cycle performance is one of the challenging issues for the biphasic electrolyte systems. For this reason, the development of more stable ion‐selective membranes, such as charge‐reinforced ion‐selective membrane,^[^ ^22^ ^]^ should be considered for the practical use of the biphasic electrolytes.

## 5 Experimental Section

### Materials

LiPF_6_ salt, EC/DMC (1/1, v/v) solvent, and 15‐crown‐5 were purchased from Enchem, Soulbrain, and Sigma‐Aldrich, respectively, to prepare 0.5 and 1 m LiPF_6_ in EC/DMC/15C5. Triethylene glycol dimethyl ether (triglyme) was purchased from Thermo Fisher Scientific to prepare 0.5 m LiPF_6_ in EC/DMC/triglyme. LiTFSI salt (Enchem), EMIM‐TFSI and HMIM‐TFSI (TCI chemicals) were purchased to prepare the electrolytes of 0.5 and 1 m LiTFSI in EMIM‐TFSI and HMIM‐TFSI. Ferrocene (Sigma‐Aldrich) was used for the calibration of the Ag/Ag^+^ reference electrode. For the preparation of Li^+^ ion‐selective membranes, Nafion membranes (N‐117, Chemours) were immersed in a 2 m LiOH aqueous solution at room temperature overnight, followed by washing with deionized water and drying in vacuum for 12 h at 60 °C. Membranes were then swelled in the mixture solvent of EC/DMC (1/1, v/v) for 3 days prior to use.

### Materials Characterizations

Raman spectra were obtained with DXR2xi (Thermo Scientific, USA) using a 532 nm laser source at 10 mW. FT‐IR spectrophotometer (TENSOR 27, Bruker, Germany) was used to analyze the solvation structures of electrolytes and to examine the ion exchange between H^+^ and Li^+^ in Nafion membranes. ^1^H and ^13^C NMR spectra (AvanceIII‐500, Bruker, Germany) were acquired using CDCl_3_ as solvent at room temperature. ICP‐AES was performed using Varian 730‐ES (Varian, Australia). ESI‐MS spectra were collected with LTQ (Thermo Finnigan, USA) using a negative scan mode. All samples were sealed in an Ar‐filled glove box prior to measurements. The viscosity of electrolytes was measured using DHR‐2 (TA instrument, US) at 25 °C.

### Electrochemical Measurements

The ionic conductivity of electrolytes was measured using a symmetric sandwich cell configuration of stainless steel | electrolyte | stainless steel in a frequency range from 50 kHz to 1 Hz with a potential amplitude of 10 mV at various temperatures. Two stainless steel electrodes were separated by a polypropylene ring with a thickness of 500 µm. The EIS measurement (SP‐150, Biologic, France) was performed in a frequency range from 1 MHz to 100 mHz with a potential amplitude of 20 mV. The electrochemical stability window of electrolytes was evaluated using linear sweep voltammetry (LSV) at a scan rate of 1 mV s^−1^. For the preparation of LiFePO_4_ olivine and layered LiCoO_2_ electrodes, active materials were mixed with carbon black (Super P) and polyvinylidene fluoride (PVdF) in a weight ratio of 8:1:1. The slurry was casted onto an Al foil current collector. Electrodes were dried in vacuum for 10 h at 120 °C prior to use. A mass loading of active materials was ≈1 mg cm^−2^. Electrochemical performances of LiFePO_4_ electrodes were evaluated in a voltage range of 3.0–4.2 V (vs Li/Li^+^ and vs Li/[Li(15C5)]^+^) using a battery measurement system (WonATech WBCS 3000). LiCoO_2_ electrodes were examined in voltage ranges of 3.0–4.2 V (vs Li/Li^+^) and 3.2–4.4 V (vs Li/[Li(15C5)]^+^), depending on the type of electrolytes. Cyclic voltammetry (CV) of Li | LiFePO_4_ cells with various electrolytes was carried out between 3.0–4.2 V (vs Li/Li^+^ and vs Li/[Li(15C5)]^+^) at a scan rate of 0.1 mV s^−1^. Home‐made cells and 2032‐type coin cells were used for biphasic and single‐phase electrolytes, respectively. In addition, H‐type cells were used for four‐electrode cells and membrane permeability tests. The redox couple of Ag/Ag^+^ (0.01 m AgNO_3_, 0.1 m tetrabutylammonium perchlorate (TBAP) in acetonitrile) was used as a reference electrode in a four‐electrode cell. The OCV values of lithium metal electrodes as a function of time were measured at 25 and 30 °C using the Ag/Ag^+^ reference electrode. The OCV profiles and the plating/stripping voltage profiles of Li/Li symmetric cells were obtained using the H‐type cells, in which the concentration of LiPF_6_ and LiTFSI were fixed at 0.5 m for each side of the biphasic electrolytes. All cells were assembled in an Ar‐filled glove box under the condition of O_2_ and H_2_O levels below 0.1 ppm.

## Conflict of Interest

The authors declare no conflict of interest.

## Supporting information

Supporting Information

## Data Availability

The data that support the findings of this study are available from the corresponding author upon reasonable request.
