# Supplementary material for: Correlation between Redox Potential and Solvation Structure in Biphasic Electrolytes for Li Metal Batteries
Source: Adv Sci (Weinh). 2022 Oct 17;9(33):2203443. doi: 10.1002/advs.202203443 (PMC9685466; doi:10.1002/advs.202203443)
Supplement: Supplementary file 1 — Supporting Information [file ADVS-9-2203443-s001.pdf]

ADVANCED SCIENCE
Open Access

Supporting Information

for *Adv. Sci.*, DOI 10.1002/advs.202203443

Correlation between Redox Potential and Solvation Structure in Biphasic Electrolytes for Li Metal Batteries

*Kyobin Park, Dong-Min Kim, Kwang-Ho Ha, Bomee Kwon, Jeonghyeop Lee, Seunghyeon Jo, Xiulei Ji and Kyu Tae Lee**

Supporting Information

Correlation between Redox Potential and Solvation Structure in Biphasic Electrolytes for Li Metal Batteries

Kyobin Park, Dong-Min Kim, Kwang-Ho Ha, Bomee Kwon, Jeonghyeop Lee, Seunghyeon Jo, Xiulei Ji, Kyu Tae Lee[*]

Supporting Discussion

Discussion S1. Potential downshift in the biphasic electrolyte cell.

A cell configuration of Li metal batteries can be theoretically described as below. This cell contains two electrolytes separated by a Li^+ ion-selective membrane.

$$\mathrm{Li} \mid \mathrm{Li^+(A)} \parallel \mathrm{Li^+(C)} \mid \mathrm{TM_xO_y} \mid \mathrm{LiTM_xO_y}$$

$LiTM_xO_y$ and TM_xO_y denote lithiated and delithiated transition metal oxide cathode materials, respectively. Li^+(A) and Li^+(C) represent Li^+ ions in the electrolytes of anode (A) and cathode (C) sides, respectively. Li indicates Li metal anode. The thermodynamic electrode potential of each half-reaction and the cell potential of the overall reaction are determined by the Nernst equation:

(i) Cathode: $\mathrm{TM_xO_y\ (s) + Li^+ + e^- \rightleftharpoons LiTM_xO_y\ (s)}$

$$E_{\mathrm{Cathode}} = E^0_{\mathrm{C}} + \frac{RT}{nF} ln \frac{a_{\mathrm{TM_xO_y}} a_{\mathrm{Li^+(C)}}}{a_{\mathrm{LiTM_xO_y}}} = E^0_{\mathrm{C}} + \frac{RT}{nF} ln\, a_{\mathrm{Li^+(C)}} \quad (1)$$

(ii) Anode: $\mathrm{Li^+ + e^- \rightleftharpoons Li\ (s)}$

$$E_{\mathrm{Li}} = E^0_{\mathrm{Li}} + \frac{RT}{nF} ln \frac{a_{\mathrm{Li^+(A)}}}{a_{\mathrm{Li}}} = E^0_{\mathrm{Li}} + \frac{RT}{nF} ln\, a_{\mathrm{Li^+(A)}} \quad (2)$$

(iii) Overall reaction:

$$E_{\mathrm{Overall}} = E_{\mathrm{Cathode}} - E_{\mathrm{Li}} = (E^0_{\mathrm{C}} - E^0_{\mathrm{Li}}) + \frac{RT}{nF} ln \frac{a_{\mathrm{Li^+(C)}}}{a_{\mathrm{Li^+(A)}}} \quad (3)$$

where E, E^0, n, F, T, R, and a represent cell potential, standard electrode potential, the number of electrons transferred in the reaction, Faraday constant, temperature, gas constant, and activity, respectively. The activity of solids was considered to be unity. For conventional Li metal batteries, cells contain a single-phase electrolyte, such as $LiPF_6$ in carbonate-based solvents. This implies that the activity of Li^+ in phase C ($a_{\mathrm{Li^+(C)}}$) is the same as that of Li^+ in phase A ($a_{\mathrm{Li^+(A)}}$) because both have the same composition of electrolytes. Accordingly, the overall cell potential is $E_{\mathrm{Cathode}} - E_{\mathrm{Li}} = (E^0_{\mathrm{C}} - E^0_{\mathrm{Li}})$, regardless of the activity of Li^+ in electrolytes. On the other hand, the overall cell potential can be modulated by the relative Li^+ activity ratio term in biphasic electrolyte cells.

Discussion S2. Formal potential of $Li/[Li(15C5)]^+$

Correlation between the redox potentials of Li/Li^+, Ag/Ag^+ and NHE references was detailed below. The redox potential of Li/Li^+ is known as follows.

$$Li/Li^+ = -3.04 \text{ V (vs. NHE)} \quad (1)$$

The redox potentials of Li/Li^+ and $Li/[Li(15C5)]^+$ with reference to the Ag/Ag^+ reference electrode are shown in Figure 1b under the condition of 1 M and 25°C.

$$Li/Li^+ = -3.2 \text{ V (vs. } Ag/Ag^+) \quad (2)$$

$$Li/[Li(15C5)]^+ = -3.48 \text{ V (vs. } Ag/Ag^+) \quad (3)$$

Combining equations (1) and (2),

$$Ag/Ag^+ = 0.16 \text{ V (vs. NHE)} \quad (4)$$

Ag/Ag^+ calibrated with reference to the Fc/Fc^+ at 25°C is shown in Figure S5.

$$Fc/Fc^+ = 0.06 \text{ V (vs. } Ag/Ag^+) \quad (5)$$

Combining equations (3) and (4), we can obtain formal potential of $Li/[Li(15C5)]^+$.

$$Li/[Li(15C5)]^+ = - 3.32 \text{ V (vs. NHE)} \quad (6)$$

Discussion S3. Liquid junction potential calculation.

Consider a double-phase electrolyte cell containing two different electrolytes on the anode side (β phase) and the cathode side (α phase). The electrochemical reaction of a cathode ($LiFePO_4$) can be expressed as:

$$\mathrm{FePO_4} + \mathrm{Li^+} + e^- \rightarrow \mathrm{LiFePO_4} \qquad (1)$$

The electrochemical potential of each species in equation (1) is expressed as:

$$\bar{\mu}_{\mathrm{FePO_4}} = \mu_{\mathrm{FePO_4}} \qquad (2)$$

$$\bar{\mu}^{\alpha}_{\mathrm{Li^+}} = \mu^{\alpha}_{\mathrm{Li^+}} + RT\ ln a^{\alpha}_{\mathrm{Li^+}} + F\phi^{\alpha} \qquad (3)$$

$$\bar{\mu}^{\alpha}_{e} = \mu^{\alpha}_{e} - F\phi^{\alpha}_{m} \qquad (4)$$

$$\bar{\mu}_{\mathrm{LiFePO_4}} = \mu_{\mathrm{LiFePO_4}} \qquad (5)$$

where $\bar{\mu}$, μ, ϕ_m, ϕ, and R stand for electrochemical potential, chemical potential, potential of electrode, potential of electrolyte, and gas constant, respectively.

The electrochemical reaction of an anode (Li metal) can be expressed as:

$$\mathrm{Li} \rightarrow \mathrm{Li^+} + e^- \qquad (6)$$

The electrochemical potential of each species in equation (6) is expressed as:

$$\bar{\mu}_{\mathrm{Li}} = \mu_{\mathrm{Li}} \qquad (7)$$

$$\bar{\mu}^{\beta}_{\mathrm{Li^+}} = \mu^{\beta}_{\mathrm{Li^+}} + RT\ ln a^{\beta}_{\mathrm{Li^+}} + F\phi^{\beta} \qquad (8)$$

$$\bar{\mu}^{\beta}_{e} = \mu^{\beta}_{e} - F\phi^{\beta}_{m} \qquad (9)$$

Thus, an overall reaction can be expressed as:

$$\mathrm{FePO_4} + \mathrm{Li}^{+(\alpha)} + e^{-(\alpha)} + \mathrm{Li} \rightarrow \mathrm{LiFePO_4} + \mathrm{Li}^{+(\beta)} + e^{-(\beta)} \qquad (10)$$

Combining equations (1)-(10), the Nernst equation can be derived as:

$$-\mathrm{F}\left(\phi^{\alpha}_{m} - \phi^{\beta}_{m}\right) = \mu_{\mathrm{LiFePO_4}} + \mu^{\beta}_{\mathrm{Li^+}} - \mu_{\mathrm{FePO_4}} - \mu^{\alpha}_{\mathrm{Li^+}} - \mu_{\mathrm{Li}} + RT\ ln\frac{a^{\beta}_{\mathrm{Li+}}}{a^{\alpha}_{\mathrm{Li+}}} + \mathrm{F}\left(\phi^{\beta} - \phi^{\alpha}\right) \qquad (11)$$

$$E = E^0 + \frac{RT}{F}\ ln\frac{a^{\alpha}_{\mathrm{Li+}}}{a^{\beta}_{\mathrm{Li+}}} + \left(\phi^{\alpha} - \phi^{\beta}\right) \qquad (12)$$

$\left(\phi^{\alpha} - \phi^{\beta}\right)$ is a liquid junction potential between two different electrolytes. For the four electrode-cell configuration (Figure 1d), difference between the redox potentials of Li/Li^+ and $LiFePO_4/FePO_4$ was E = 3.69 V, which is 0.02 V higher than the cell potential of $[Li(15C5)]^+/Li^+$ ($E^0 + \frac{RT}{F}\ ln\frac{a^{\alpha}_{\mathrm{Li+}}}{a^{\beta}_{\mathrm{Li+}}}$ = 3.67 V). Therefore, the liquid junction potential $\left(\phi^{\alpha} - \phi^{\beta}\right)$ of the $[Li(15C5)]^+/Li^+$ cell is 0.02 V

Discussion S4. Relative Li^+ activity ratio calculation in $[Li(15C5)]^+/Li^+$ cell.

Consider 1 M $LiPF_6$ in EC/DMC and 1 M $LiPF_6$ in EC/DMC/15C5 as phase α and phase β, respectively, at 25 ℃ in Figure 1b. The redox potentials of Li metal electrodes in phase α and phase β can be expressed by the Nernst equation in the form of:

$$E_\alpha = E^0 + \frac{RT}{F}\ln a^\alpha_{Li+} \qquad (1)$$

$$E_\beta = E^0 + \frac{RT}{F}\ln a^\beta_{Li+} \qquad (2)$$

The potential difference between two Li metal electrodes in α and β at 25 ℃ is expressed as:

$$\Delta E = E_\beta - E_\alpha = \frac{RT}{F}\ln\frac{a^\beta_{Li+}}{a^\alpha_{Li+}} = 0.0591\log\frac{a^\beta_{Li+}}{a^\alpha_{Li+}} \qquad (3)$$

The electrode potentials of Li metal in α and β (E_α and E_β) measured using the Ag/Ag^+ reference electrode were -3.20 V and -3.48 V (vs. Ag/Ag^+), respectively. This implies that $\Delta E = 0.0591\log\frac{a^\beta_{Li+}}{a^\alpha_{Li+}} = 0.28\ V$, revealing that the Li^+ activity in the conventional carbonate-based electrolyte without 15C5 ($a_{Li^+(C)}$) was approximately 5.5×10^4 times higher than that in the electrolyte containing 15C5 ($a_{Li^+(A)}$).

Discussion S5. Role of the complexation effect in Li^+ activity.

Gibbs free energy of chelation can be expressed as $\Delta G = \Delta H - T\Delta S$, where the chelating effect contributes to increasing entropy (ΔS) and the macrocyclic effect gives rise to an increase in entropy (ΔS) and a decrease in enthalpy (ΔH).[1] Therefore, both effects improve the thermodynamic stability of the chelating structures, such as $[Li(15C5)]^+$ and $[Li(triglyme)]^+$ ($\Delta G < 0$). Moreover, the macrocyclic effect suggests that interaction between Li^+ and cyclic 15C5 is stronger than that between Li^+ and linear triglyme.

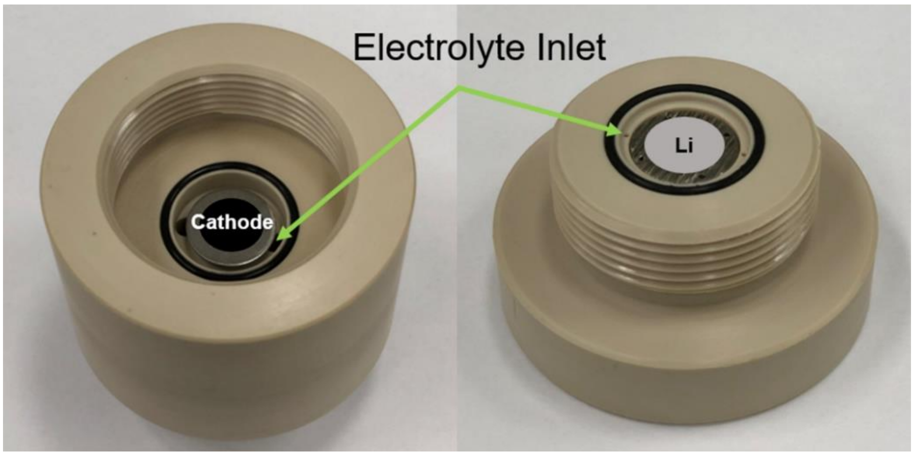

Figure S1. Photograph of home-made cell components for bilayer electrolytes.

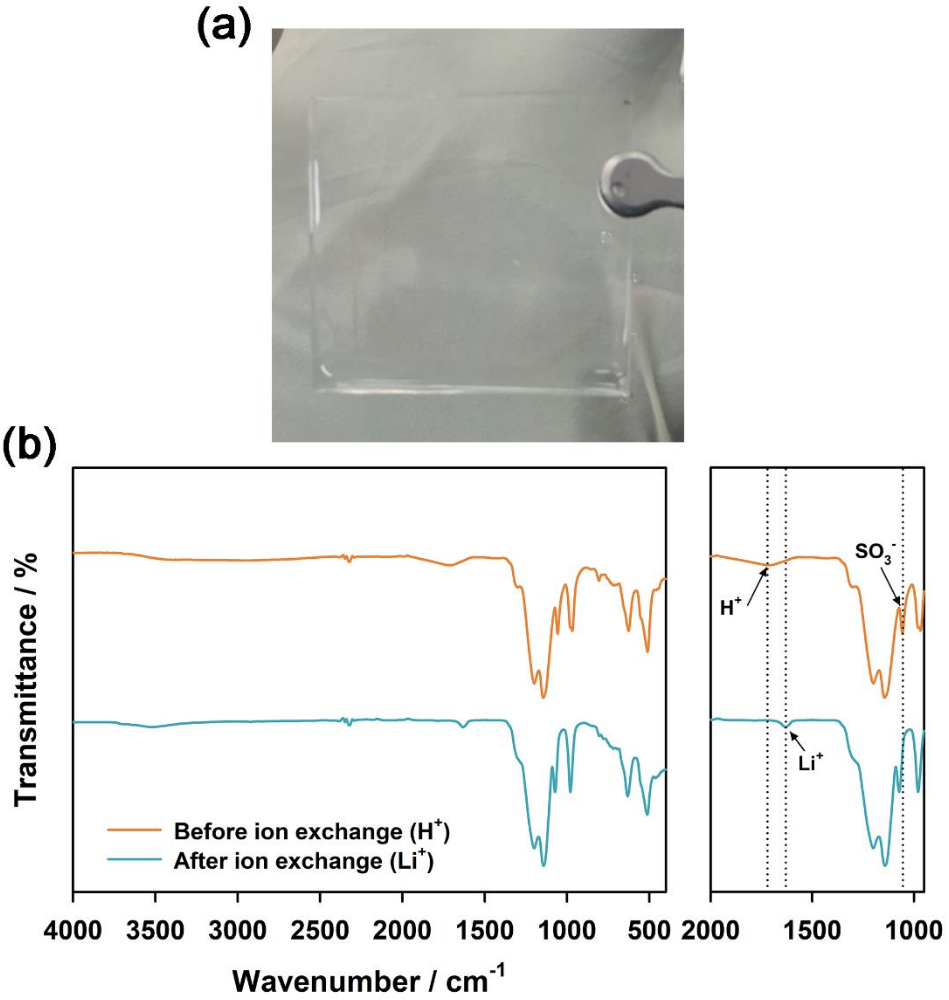

Figure S2. (a) Photograph of the Li^+ ion-selective Nafion membrane obtained after ion exchange between H^+ and Li^+. (b) FT-IR spectra of the Li^+ ion-selective Nafion membrane before and after ion exchange between H^+ and Li^+.

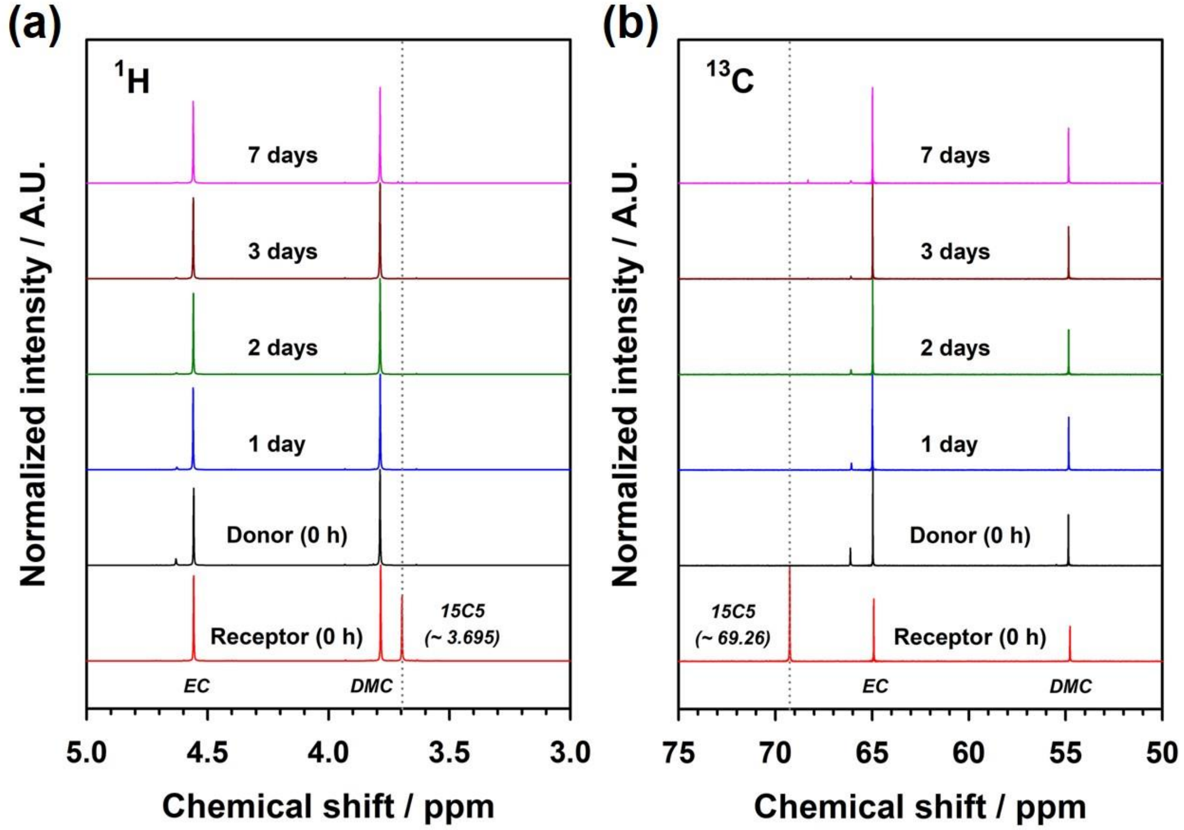

Figure S3. Membrane permeability to 15C5. (a) ^{1}H and (b) ^{13}C NMR spectra of solutions in the donor chamber of the 2-chamber side diffusion cell separated with the Li^{+} ion-selective Nafion membrane. Solutions were retrieved from the donor chamber after various periods of time. The donor chamber was filled with 0.5 M $LiPF_6$ in EC/DMC, whereas the receptor one was filled with 0.5 M $LiPF_6$ in EC/DMC/15C5.

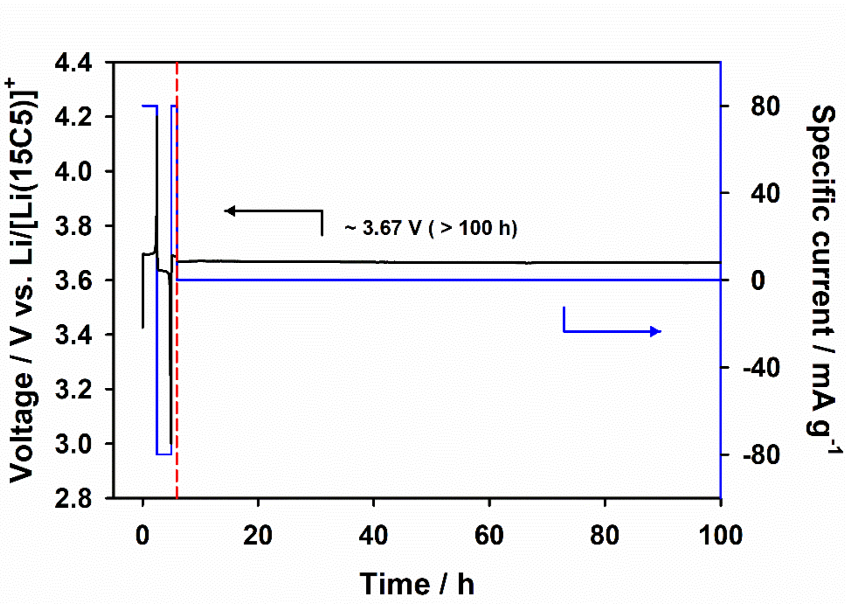

Figure S4. OCV profile of Li | 0.5 M $LiPF_6$ in EC/DMC/15C5 ‖ 0.5 M $LiPF_6$ in EC/DMC | $LiFePO_4$ at the SOC of 50% and 30 °C after one cycle at a 0.5C rate.

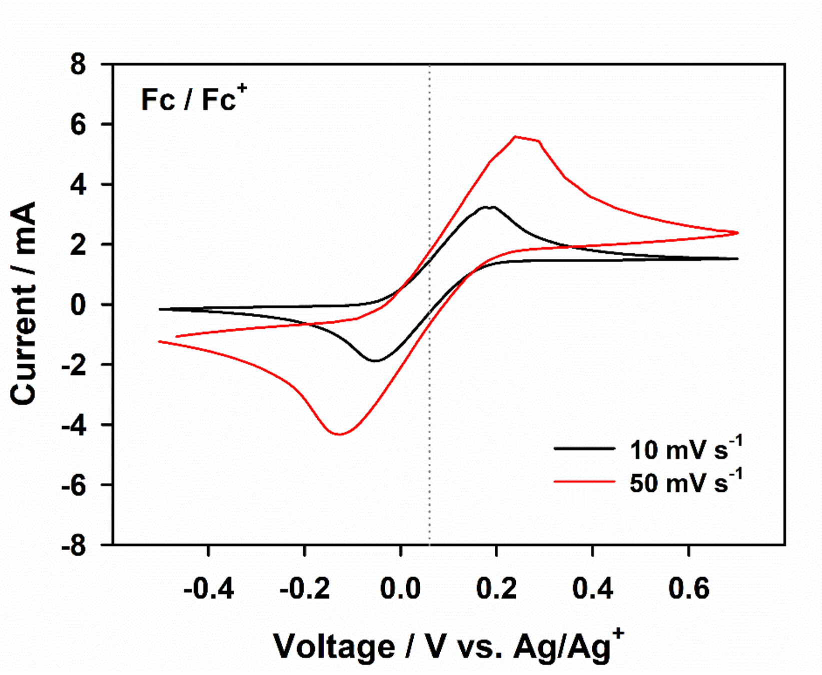

Figure S5. Cyclic voltammograms of the Fc/Fc^{+} redox couple in the electrolyte of 1 M $LiPF_6$ EC/DMC at scan rates of 10 mV s^{-1} and 50 mV s^{-1} using a three-electrode cell configuration consisting of glassy carbon working electrode, Pt-wire counter electrode, and Ag/Ag^{+} reference electrode at 25 °C.

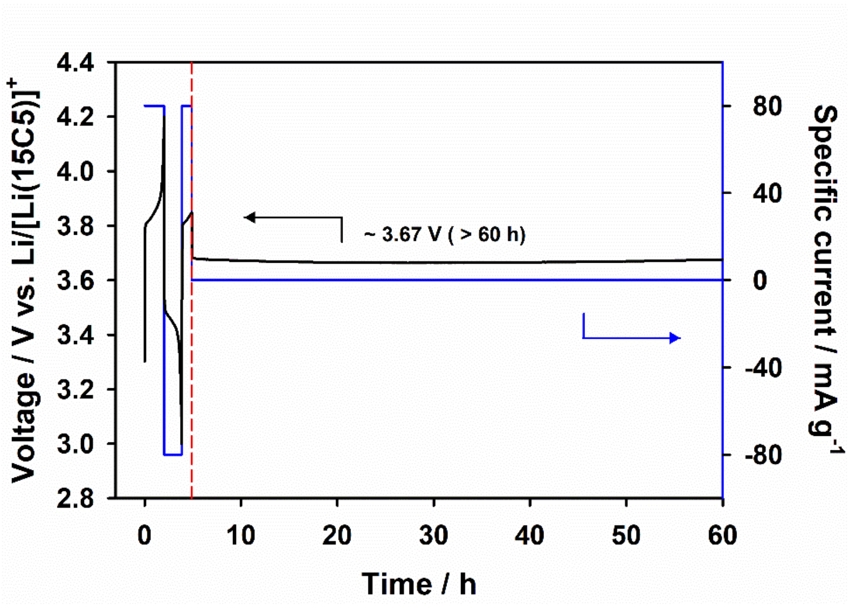

Figure S6. OCV profile of Li | 1 M $LiPF_6$ in EC/DMC/15C5 ‖ 1 M $LiPF_6$ in EC/DMC | $LiFePO_4$ at the SOC of 50% and 25 °C after one cycle at a 0.5C rate.

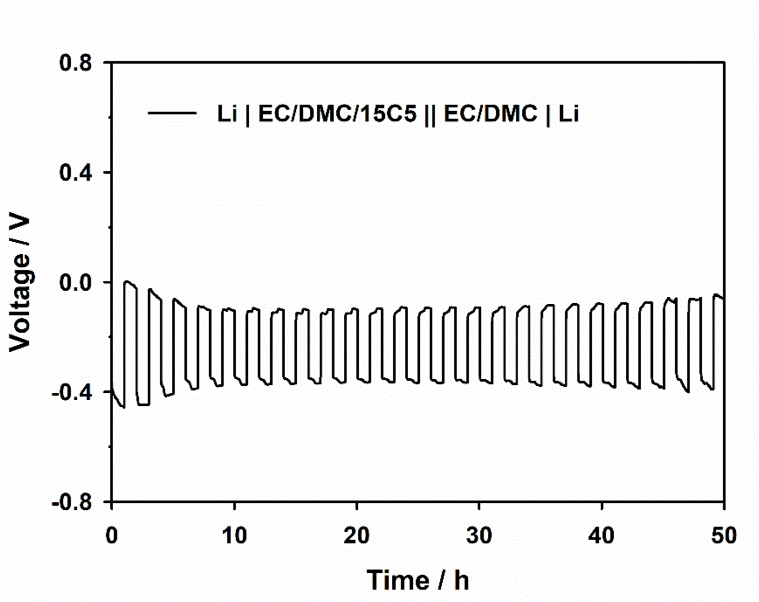

Figure S7. Voltage profile of a symmetric cell for Li | 0.5 M $LiPF_6$ in EC/DMC/15C5 ‖ 0.5 M $LiPF_6$ in EC/DMC | Li under the condition of an areal capacity of 0.5 mA h cm^{-2} at a current density of 0.5 mA cm^{-2}.

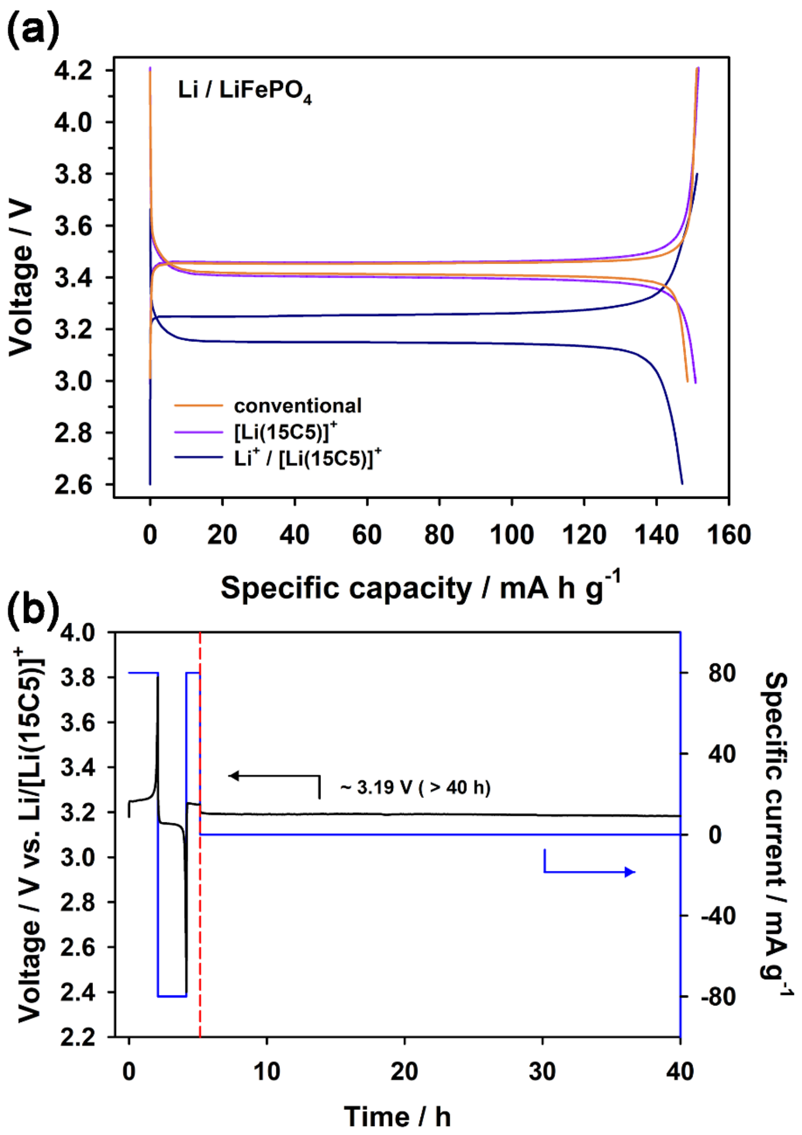

Figure S8. (a) Voltage profiles of (i) conventional cell (Li | 1 M $LiPF_6$ in EC/DMC | $LiFePO_4$), (ii) $[Li(15C5)]^+$ cell (Li | 0.5 M $LiPF_6$ in EC/DMC/15C5 | $LiFePO_4$), and (iii) $Li^+/[Li(15C5)]^+$ cell (Li | 0.5 M $LiPF_6$ in EC/DMC ‖ 0.5 M $LiPF_6$ in EC/DMC/15C5 | $LiFePO_4$) at a 0.5C rate and 30 °C. **(b)** OCV profile of Li | 0.5 M $LiPF_6$ in EC/DMC ‖ 0.5 M $LiPF_6$ in EC/DMC/15C5 | $LiFePO_4$ at the SOC of 50% and 30 °C. OCV profiles were obtained after one cycle at a 0.5C rate.

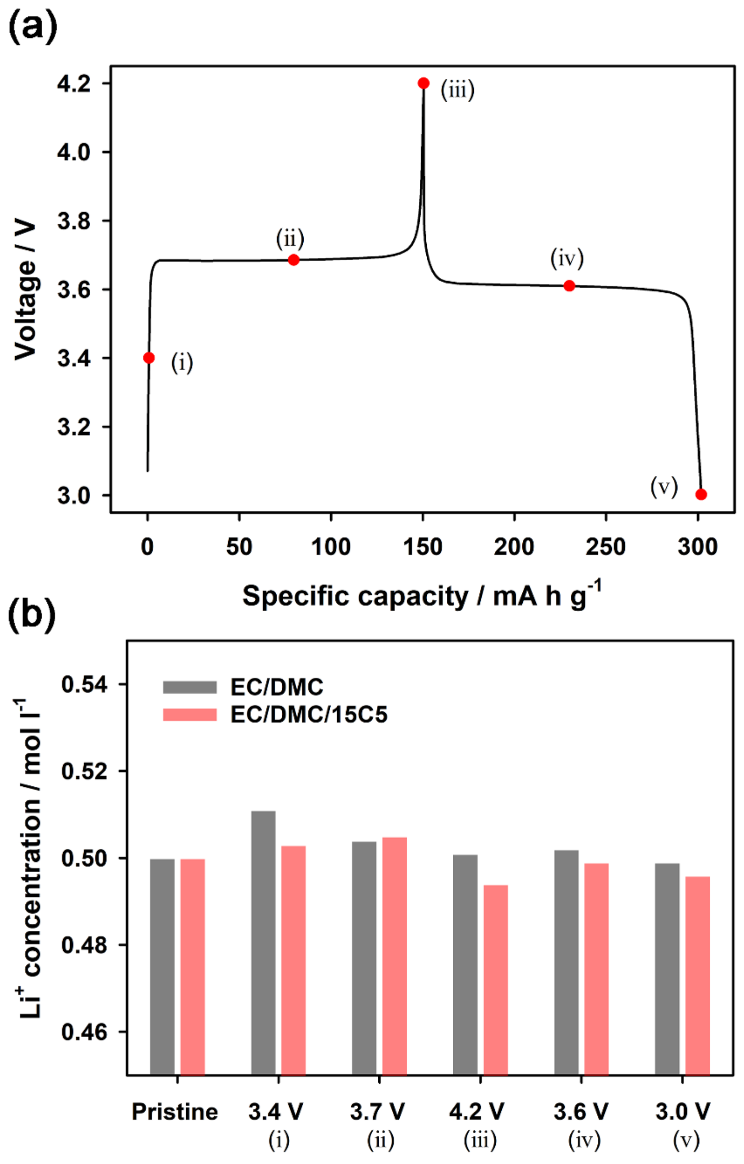

Figure S9. (a) Voltage profile of Li(15C5)]$^{+}$/Li^{+} cell for $LiFePO_4$ at a 0.5C rate and 30 °C and (b) Li^{+} concentrations at the anode side (0.5 M $LiPF_6$ in EC/DMC/15C5, 2/2/1) and the cathode side (0.5 M $LiPF_6$ in EC/DMC) solutions retrieved at various charge/discharge states indicated in the voltage profile of (a).

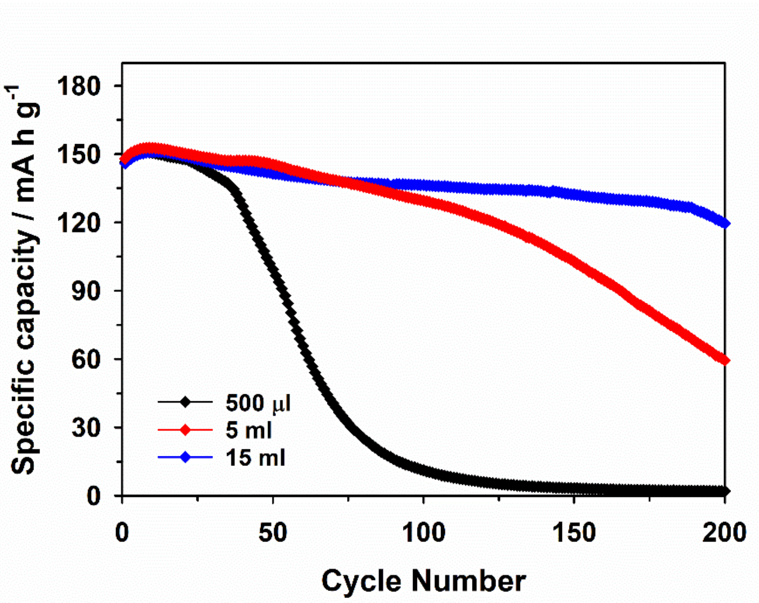

Figure S10. Cycle performance of $[Li(15C5)]^{+}/Li^{+}$ cells consisting of $LiFePO_4$ cathode and Li metal anode at a 4C rate for various amounts of electrolyte per *ca.* 1 mg cm^{-2} of $LiFePO_4$.

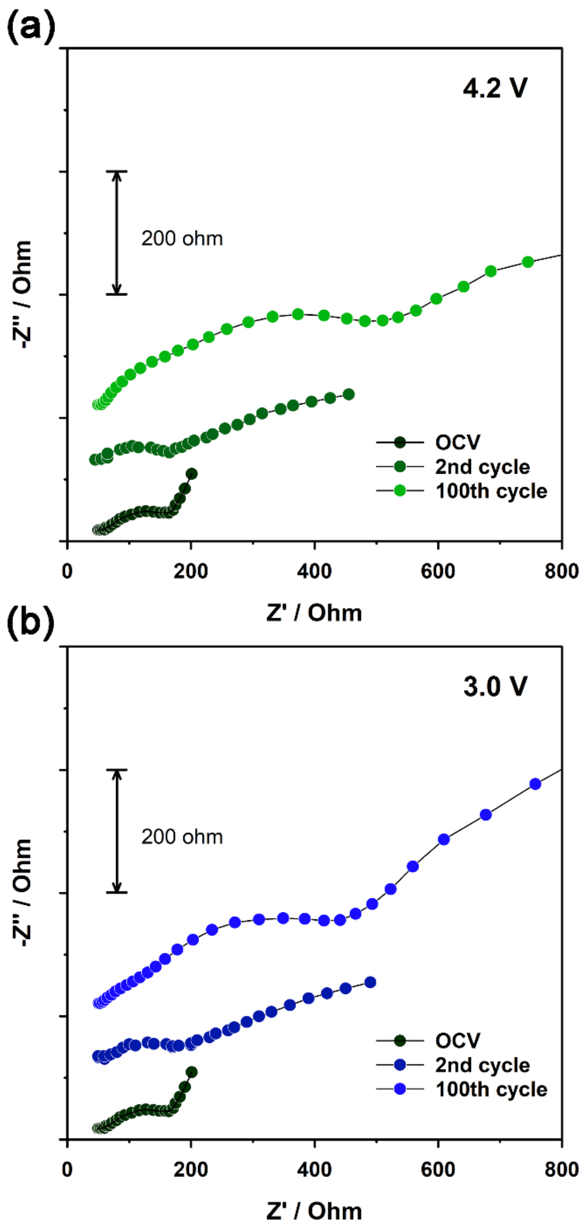

Figure S11. Nyquist plots of $[Li(15C5)]^+/Li^+$ cell for $LiFePO_4$ for various cycle numbers at fully (a) charged (4.2 V vs. Li/Li^+) and (b) discharged (3.0 V vs. Li/Li^+) states.

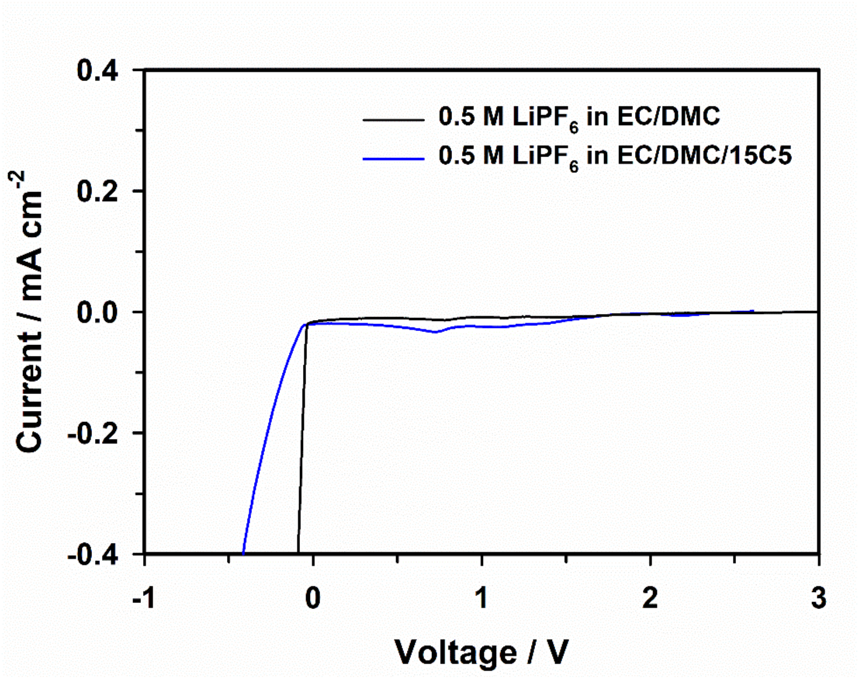

Figure S12. LSV profiles of 0.5 M $LiPF_6$ in EC/DMC (1/1, v/v) and 0.5 M $LiPF_6$ in EC/DMC/15C5 (2/2/1, v/v/v) at a scan rate of 1 mV s^{-1}. Cu foil was used as a working electrode. Li metal was used as counter and reference electrodes.

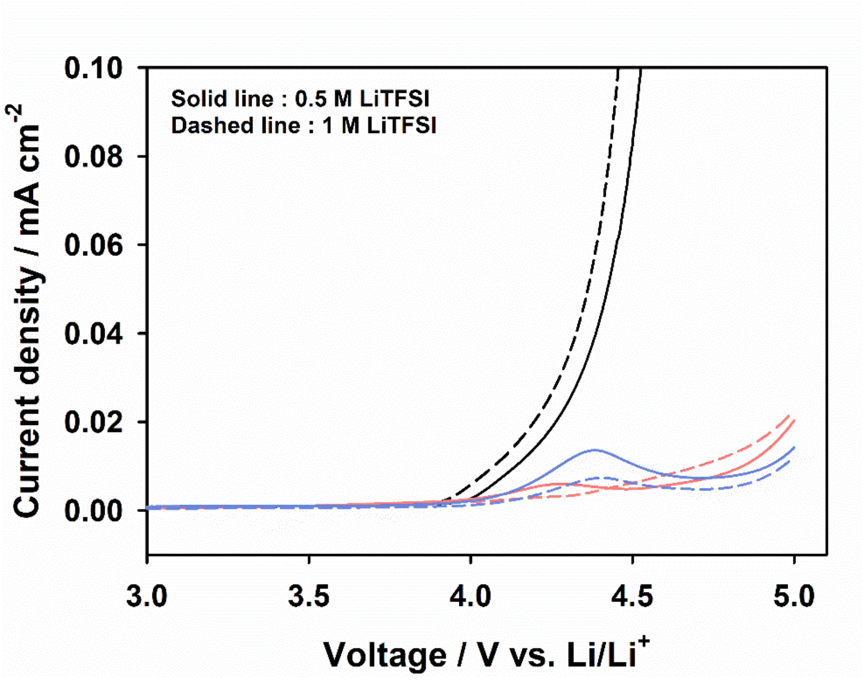

Figure S13. LSV profiles of 0.5 M (solid line) and 1 M (dashed line) LiTFSI in EC/DMC (1/1, v/v, black line), EMIM-TFSI (red line), and HMIM-TFSI (blue line) at a scan rate of 1 mV s^{-1}. Al foil was used as a working electrode. Li metal was used as counter and reference electrodes.

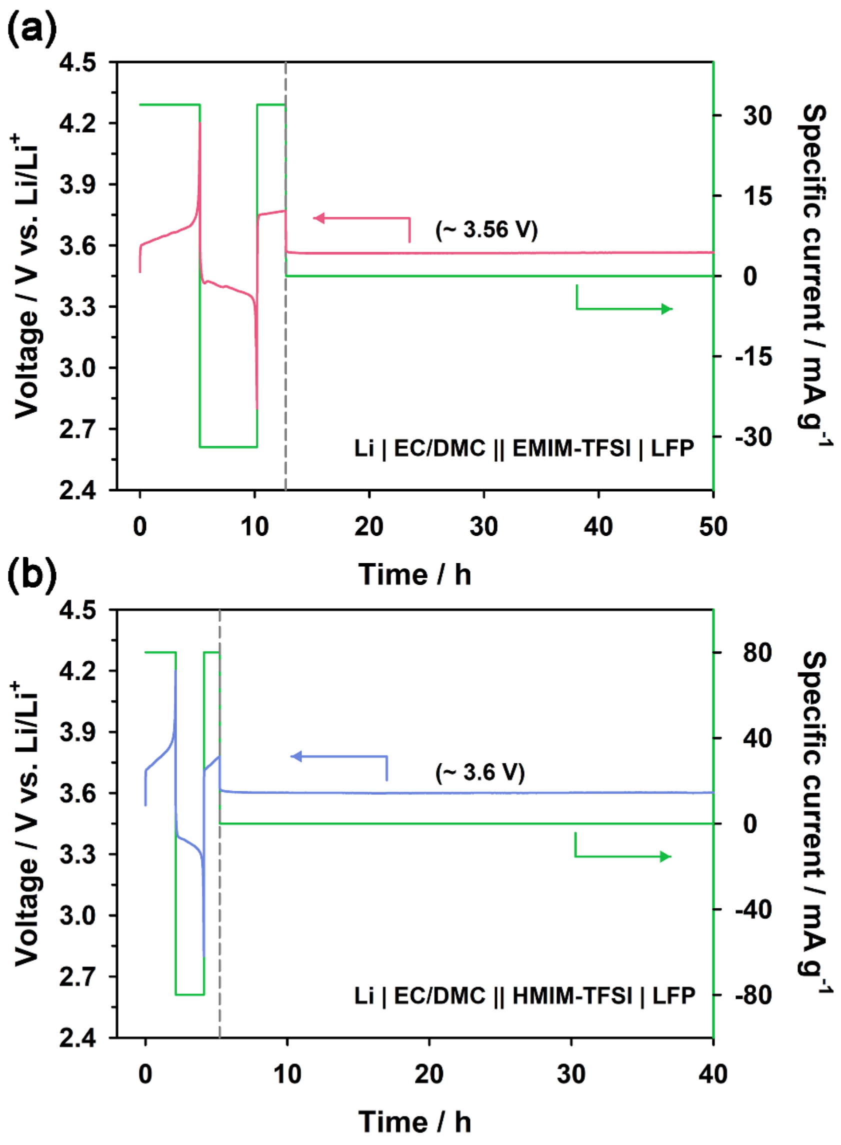

Figure S14. OCV profiles of (a) Li | 1 M LiTFSI in EC/DMC ǁ 1 M LiTFSI in EMIM-TFSI | $LiFePO_4$ and (b) Li | 1 M LiTFSI in EC/DMC ǁ 1 M LiTFSI in HMIM-TFSI | $LiFePO_4$ at the SOC level of 50 % and 30 °C. OCV profiles were obtained after one cycle at (a) 0.2C and (b) 0.5C rates.

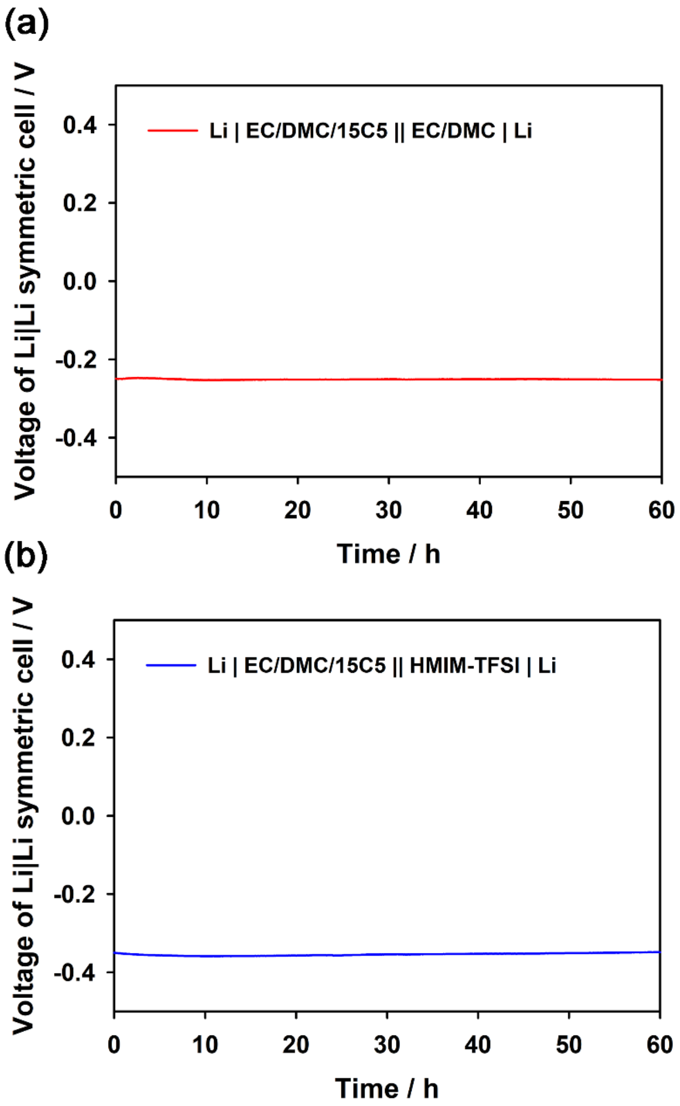

Figure S15. OCV profiles of (a) Li | 0.5 M $LiPF_6$ in EC/DMC/15C5, 2/2/1 ‖ 0.5 M $LiPF_6$ in EC/DMC | Li and (b) Li | 0.5 M LiTFSI in EC/DMC/15C5 ‖ 0.5 M LiTFSI in HMIM-TFSI | Li.

Table S1. Li^+ concentration of solutions in the donor and receptor chambers of the 2-chamber side diffusion cell separated with the Li^+ ion-selective Nafion membrane. Solutions were retrieved from chambers after various periods of time. The donor chamber was filled with 0.5 M $LiPF_6$ in EC/DMC, whereas the receptor one was filled with 0.5 M $LiPF_6$ in EC/DMC/15C5. Li^+ concentration of solutions was measured using ICP-AES.

Storage time	Li^+ concentration in the donor chamber (mol l^{-1})	Li^+ concentration in the receptor chamber (mol l^{-1})
Pristine	0.5	0.5
1 day	0.506	0.501
2 days	0.524	0.497
3 days	0.523	0.499
7 days	0.515	0.498

Table S2. Values of specific capacity and average operating voltage used to calculate the energy density of various cathode materials shown in Figure 3b. These values were obtained from the other literatures.[2-3]

Cathode materials	Specific capacity ($mA\ h\ g^{-1}$)	Average Operating Voltage (V)
$LiCo_2O_4$	84	4
$LiMn_2O_4$	120	4.1
$LiNi_{0.5}Mn_{1.5}O_4$	125	4.7
$LiCoPO_4$	125	4.2
$LiFePO_4$	165	3.4
$LiFeSO_4F$	120	3.7
$LiVPO_4F$	129	4.2
$LiMnO_2$	140	3.3
$LiCoO_2$	190 (3.0 ~ 4.45 V)	3.9
$LiNi_{0.33}Mn_{0.33}Co_{0.33}O_2$	185 (3.0 ~ 4.5 V)	3.8
$LiNi_{0.8}Co_{0.15}Al_{0.05}O_2$	210 (3.0 ~ 4.4 V)	3.8

Table S3. Physiochemical properties of 0.5 M $LiPF_6$ EC/DMC/15C5 (2/2/x, v/v/v) for various molar ratios of 15C5 to Li^+.

**Material property**	**15C5:Li^+ = 0:1**	**15C5:Li^+ = 1:1**	**15C5:Li^+ = 2:1**	**15C5:Li^+ = 3:1**	**15C5:Li^+ = 4:1**	**15C5:Li^+ = 5:1**
Volume fraction of 15C5 (x)	0	0.45	1.01	1.74	2.71	4.10
Activation energy (kJ mol^{-1})	10.31	11.29	12.21	14.21	16.64	20.30
Viscosity (mPa s)	2.27	2.05	2.70	3.32	5.02	9.42

Table S4. Raman vibration frequencies of 0.5 M $LiPF_6$ EC/DMC/15C5 electrolytes.[4-5]

**Frequency (cm^{-1})**	**Assignments**
718	EC (C=O)
730	EC (C=O Li^+)
742	PF_6^-
830	15C5 (C-O)
875	Li-O
894	EC (C-O)
905	EC (C-O Li^+)
916	DMC (C-O)

Table S5. FT-IR vibration frequencies of 0.5 M $LiPF_6$ EC/DMC/15C5 electrolytes.[6]

**Frequency (cm^{-1})**	**Assignments**
843	PF_6^-
1070	EC (C-O)
1155	EC (CH_2)
1390	EC (CH_2)
1481	EC (CH_2)
1723	DMC (C=O Li^+)
1772	EC (C=O Li^+)
1801	EC (C=O)

Table S6. ^{13}C NMR chemical shifts of 0.5 M $LiPF_6$ EC/DMC/15C5 electrolytes.

**Chemical shift (ppm)**	**Assignments**
54.86	DMC (CH_2)
64.77	EC (CH_2)
155.64	EC (C=O)
156.26	DMC (C=O)

Supplementary References

[1] F. P. Hinz, D. W. Margerum, *Inorg. Chem.* **1974**, *13*, 12.

[2] N. Nitta, F. Wu, J. T. Lee, G. Yushin, *Mater. Today* **2015**, *18*, 5.

[3] J. Zheng, S. Myeong, W. Cho, P. Yan, J. Xiao, C. Wang, J. Cho, J.-G. Zhang, *Adv. Energy Mater.* **2017**, *7*, 6.

[4] G. Bouteau, A. N. Van-Nhien, M. Sliwa, N. Sergent, J.-C. Lepretre, G. Gachot, I. Sagaidak, F. Sauvage, *Sci. Rep.* **2019**, *9*, 1.

[5] D. Brouillette, D. E. Irish, N. J. Taylor, G. Perron, M. Odziemkowski, J. E. Desnoyers, *Phys. Chem. Chem. Phys.* **2002**, *4*, 24.

[6] Y. Ikezawa, H. Nishi, *Electrochim. Acta* **2008**, *53*, 10.
